# Global reconstruction of life‐history strategies: A case study using tunas

**DOI:** 10.1111/1365-2664.13327

**Published:** 2019-02-01

**Authors:** Cat Horswill, Holly K. Kindsvater, Maria José Juan‐Jordá, Nicholas K. Dulvy, Marc Mangel, Jason Matthiopoulos

**Affiliations:** ^1^ Institute of Biodiversity, Animal Health & Comparative Medicine University of Glasgow Glasgow UK; ^2^ Department of Zoology University of Cambridge Cambridge UK; ^3^ Department of Ecology, Evolution, and Natural Resources Rutgers University New Brunswick New Jersey; ^4^ AZTI Pasaia Gipuzkoa Spain; ^5^ Earth to Ocean Research Group Department of Biological Sciences Simon Fraser University Burnaby British Columbia Canada; ^6^ Theoretical Ecology Group Department of Biology University of Bergen Bergen Norway; ^7^ Institute of Marine Sciences Department of Applied Mathematics University of California Santa Cruz California

**Keywords:** Bayesian imputation, data limited, demography, fecundity, life‐history theory, missing data, principal market tuna, Scombridae

## Abstract

1. Measuring the demographic parameters of exploited populations is central to predicting their vulnerability and extinction risk. However, current rates of population decline and species loss greatly outpace our ability to empirically monitor all populations that are potentially threatened.
2. The scale of this problem cannot be addressed through additional data collection alone, and therefore it is a common practice to conduct population assessments based on surrogate data collected from similar species. However, this approach introduces biases and imprecisions that are difficult to quantify. Recent developments in hierarchical modelling have enabled missing values to be reconstructed based on the correlations between available life‐history data, linking similar species based on phylogeny and environmental conditions.
3. However, these methods cannot resolve life‐history variability among populations or species that are closely placed spatially or taxonomically. Here, theoretically motivated constraints that align with life‐history theory offer a new avenue for addressing this problem. We describe a Bayesian hierarchical approach that combines fragmented, multispecies and multi‐population data with established life‐history theory, in order to objectively determine similarity between populations based on trait correlations (life‐history trade‐offs) obtained from model fitting.
4. We reconstruct 59 unobserved life‐history parameters for 23 populations of tuna that sustain some of the world's most valuable fisheries. Testing by cross‐validation across different scenarios indicated that life‐histories were accurately reconstructed when information was available for other populations of the same species. The reconstruction of several traits was also accurate for species represented by a single population, although credible intervals increased dramatically.
5. *Synthesis and applications*. The described Bayesian hierarchical method provides access to life‐history traits that are difficult to measure directly and reconstructs missing life‐history information useful for assessing populations and species that are directly or indirectly affected by human exploitation of natural resources. The method is particularly useful for examining populations that are spatially or taxonomically similar, and the reconstructed life‐history strategies described for the principal market tunas have immediate application to the world‐wide management of these fisheries.

## Abstract 2

The described Bayesian hierarchical method provides access to life‐history traits that are difficult to measure directly and reconstructs missing life‐history information useful for assessing populations and species that are directly or indirectly affected by human exploitation of natural resources. The method is particularly useful for examining populations that are spatially or taxonomically similar, and the reconstructed life‐history strategies described for the principal market tunas have immediate application to the world‐wide management of these fisheries.

## 1 INTRODUCTION

Monitoring a population's status and risk of extinction requires detailed knowledge of life‐history characteristics, including survival, growth and reproduction (Pearson et al., 2014; Salguero‐Gómez et al., 2016; Winemiller, 2005). However, our understanding of these traits is fragmented, error‐prone and incomplete for many species (Myers, Mittermeier, Mittermeier, da Fonseca, & Kent, 2000). For example, more than 10% of the 87,967 animals, plants and fungi assessed by the International Union for Conservation of Nature (IUCN) are classified as Data Deficient, such that their status and vulnerability cannot be determined (IUCN, 2017). Consequently, identifying reliable ways to detect declines and assess the extinction risk of data‐limited populations has been highlighted as a key ecological problem facing policy makers (Kindsvater et al., 2018; Sutherland et al., 2006).

One approach of assessing data‐limited populations involves judiciously filling‐in missing values of life‐history parameters with surrogate data from similar species or other populations of the same species. However, using surrogate parameters concurrently with observed life‐history traits neglects the trade‐offs, that is, budgetary compromises, that connect the different aspects of a population's demography, constraining the range of possible life‐history strategies that can evolve (Stearns, 1992). The biases and imprecisions generated by employing this approach are difficult to quantify, creating a considerable margin for parameter misuse and population mismanagement. The process of using surrogate life‐history values to overcome missing data has become increasingly formalized with the widespread availability of meta‐analyses for key parameters (e.g. Denney, Jennings, & Reynolds, 2002; Hutchings, Myers, Garcia, Lucifora, & Kuparinen, 2012; Thorson, Taylor, Stewart, & Punt, 2014). Predictive approaches have also been developed to estimate missing values across large groups of species based on correlations between available life‐history data (Freckleton & Jetz, 2009; Jetz & Freckleton, 2015; Thorson, Munch, Cope, & Gao, 2017). These studies predict life‐history parameters by grouping species based on phylogeny and fine‐scale environmental determinants of life‐history traits, such as temperature.

Commercial fisheries exploit populations at rates that greatly outpace our ability to accurately assess them. Populations with missing life‐history data are particularly problematic in this context because, by definition, the data necessary to develop evidence‐based management and policy action are incomplete. At present, less than 20% of the global fish catch originates from populations that are managed by formal population assessment (Costello, 2012), and therefore, threats from over‐exploitation are rarely identified before species have suffered large population declines (Burgess, Polasky, & Tilman, 2013). Correlative approaches have been used to estimate specific life‐history traits for data‐limited fish species (Beverton, 1992; Hordyk, Ono, Sainsbury, Loneragan, & Prince, 2014; Nadon & Ault, 2016), incorporating phylogenetic and environmental determinants of traits to access large numbers of species (Thorson et al., 2017). However, phylogeny becomes less informative when considering population‐level strategies, as opposed to species‐level strategies, and when examining species within the same genus. Furthermore, populations that inhabit large geographic areas and migrate over broad latitudinal gradients preclude a straightforward assignment of fine‐scale environmental determinants for life‐history strategy. Predictive approaches informed by theoretically motivated constraints that align with life‐history theory (Kindsvater et al., 2018) offer a new avenue for providing informative distinctions between populations that are closely placed taxonomically and spatially.

**Figure 1:**
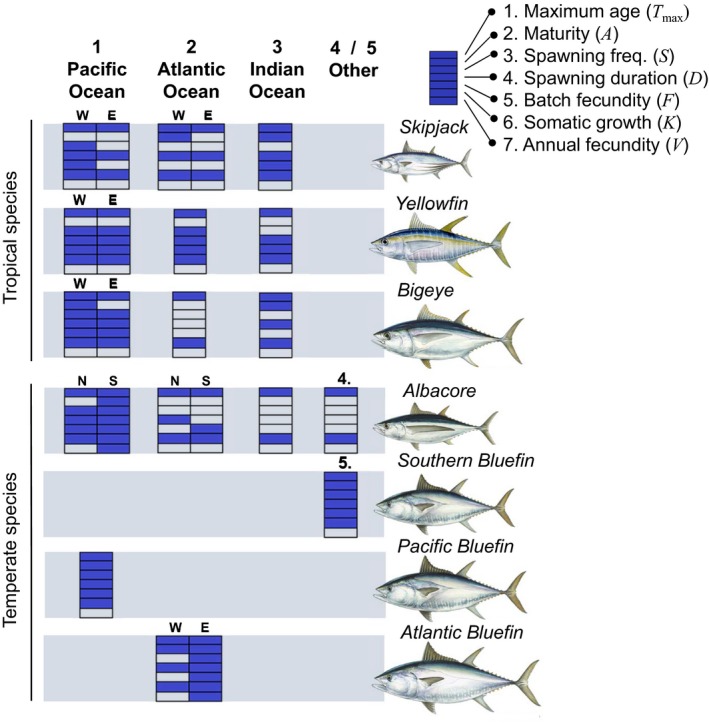
Availability of data for each life‐history trait for the seven species of principal market tunas grouped by population, species and habitat. Shaded blocks indicate that life‐history data are available in the original dataset for that population. Each column reflects a major ocean region and population; 1. Pacific Ocean, 2. Atlantic Ocean, 3. Indian Ocean, 4. Mediterranean Sea and 5. Southern Ocean, where two populations are present within one region, its location is indicated above the bar: N = north, S = south, W = west and E = east. Fish pictures from Diane Rome Peebles. Species listed in ascending order of growth rates (*k*, Juan‐Jordá et al., 2016). Population‐specific survival rates are derived from maximum age (*T*_max_, Equation (1)). Supporting Information Table S1 shows number of studies for each population per trait

The seven principal market tunas (skipjack tuna – *Katsuwonus pelamis*, yellowfin tuna – *Thunnus albacares*, bigeye tuna – *Thunnus obesus*, albacore tuna – *Thunnus alalunga*, Pacific bluefin tuna – *Thunnus orientalis*, Atlantic bluefin tuna – *Thunnus thynnus*, Southern bluefin tuna – *Thunnus maccoyii*) are categorized into 23 highly migratory stocks, or populations, with ocean‐scale distributions. These populations sustain some of the largest and most valuable fisheries in the world (Juan‐Jordá, Mosqueira, Cooper, Freire, & Dulvy, 2011), totalling a sales value of US 41 billion (FAO, 2016; MacFadyen, 2016) and accounting for approximately 9% of global fish catches (Costello, 2012; FAO, 2014). However, these populations have also experienced long‐term declines in adult biomass since the 1950s (Juan‐Jordá et al., 2011), such that 43% are currently overfished with biomass levels below management recommendations (ISSF, 2018). Furthermore, population‐specific life‐history data are missing for more than a third (36%) of maturity and fecundity parameters (Figure 1; see Supporting Information Table S1). For example, age of maturity and annual fecundity are poorly resolved, and are only reported for 10 and two of the 23 populations respectively. Consequently, population assessments often rely on age of maturity estimates from other populations, and spawning stock biomass is generally used as an index of fecundity when fitting stock recruitment models (e.g. ISC, 2016, Rice, Harley, Davies, & Hampton, 2014).

Here, we develop a Bayesian hierarchical approach that considers a multi‐population, multispecies dataset and reconstructs a full life‐history strategy for the 23 populations of principal market tuna that are recognized globally. We employ a multivariate approach that determines the direction and strength of life‐history trade‐offs among traits in order to resolve the similarity between species. This framework provides access to traits that are difficult to measure directly, such as age of maturity and annual fecundity. Furthermore, by considering multiple sources of information and including error in the observed data, it also generates a measure of uncertainty associated with each parameter. The model outlined here is applied to tunas; however, the approach could be applied to a wide range of taxa and we explore the predictive capacity of the method through a series of cross‐validation experiments.

## 2 MATERIALS AND METHODS

### 2.1 Life‐history traits for the principal market tunas

We considered a seven trait life‐history strategy for the principal market tunas. Data on six of these traits were collated from Juan‐Jordá, Mosqueira, Freire, Ferrer‐Jordá, and Dulvy (2016). As the principal market tunas are sexually dimorphic, we removed trait estimates based on males only. Traits included adult survival (*Φ*) derived from maximum age (*T*_max_; Equation (1)); age of maturity (*A*, age that 50% of the sampled individuals are mature); spawning frequency (*S*, number of days between each batch of eggs, oocytes, being produced); spawning duration (*D*, proportion of the year that the population is actively spawning); absolute batch fecundity (*F*, mean number of oocytes produced per individual per batch); and somatic growth rate (*K*) from the von Bertalanffy growth function. Three additional studies on batch fecundity published after the original dataset was compiled were also included in order to bolster populations that were missing data across the four fecundity traits (Supporting Information Table S1). To resolve a full set of fecundity parameters, information on the seventh life‐history trait, annual fecundity per capita (*V*, mean number of oocytes produced per individual per year), was also included for the two populations with published estimates (Figure 1; Supporting Information Table S1). Finally, as broad similarities in life‐history strategy can be drawn between species of tuna that inhabit the same biome (Juan‐Jordá, Mosqueira, Freire, & Dulvy, 2013, 2015), each population was assigned a binary habitat variable (*H*) delineating tropical or temperate preference (Figure 1). To promote convergence across life‐history parameters with different scales (Congdon, 2003), each trait was standardized to a mean of 0 and a standard deviation of 1.

Rates of adult survival (*Ф*) were based on the following estimation of natural mortality (*M*) from maximum age (*T*_max_), where 4.3 is an estimated constant (Kenchington, 2014):

(1) $$M=\frac{4.3}{T_{\max}},\;\Phi =e^{-M}$$

Mortality estimates that are based on maximum age reportedly perform better than those based on other parameters, such as body size (Then et al., 2015). Using a maximum age estimator also provided the largest sample size for rates of adult survival from the starting dataset, and allowed the estimation of this trait to be independent from metrics of body growth that would be used simultaneously to impute mortality in later analysis. Natural mortality is more commonly included as a demographic trait in fisheries assessment, compared to survival. However, we include the converted value of adult survival in order to demonstrate the wider application of this method to alternative systems where viability assessments are based on this trait. Varying amounts of life‐history information were available for each population (Juan‐Jordá et al., 2013; Figure 1; Supporting Information Table S1), and few studies provided information on more than one or two life‐history traits. Thus, examination of trade‐offs at the individual‐study level was not possible.

### 2.2 Model of life‐history traits

The Bayesian hierarchical model comprised a demographic component with parameters describing species‐specific relationships between life‐history traits, and an observation component that integrated observed data for each population with trait‐specific uncertainty associated with measurement error and process variability in the data (e.g. spatial and temporal variation). The code and data file for the model are provided in Appendices S1 and S2 of the Supporting Information, and key model parameters are listed in Supporting Information Table S2. All models were implemented in JAGS (v. 4.3.0) via the “jagsUI” library (v 1.4.9) for program r (v. 3.4.1). Models were fitted by running three Monte Carlo Markov chains (MCMC) for 1.5 × 10^7^ iterations and retaining every 200th step in order to increase the effective MCMC sample size for the same amount of computer memory. The first 5,000 MCMC draws were removed as burn‐in, and each chain was initialized at different points in the parameter space. Convergence of the chains was confirmed using the Brooks–Gelman–Rubin diagnostic tool (all values $\hat{r}$ ≤ 1.01) and the effective sample size for each parameter (we required this to be >500).

The demographic component of the model (Equation (2)) contained a function for each life‐history trait. Each trait was modelled in relation to a binary habitat term (*H*) delineating tropical and temperate species, and two random‐effect terms (*γ* and *ω*) that represent the correlated residuals (i.e. trade‐offs, Charnov, 1993) between traits at the species‐and population‐level, respectively:

(2) $$\begin{array}{cc} \phi_{i,j} & =\psi_{\phi }H_{i}+\gamma_{\phi ,j}+\omega_{\phi ,i}\\ a_{i,j} & =\psi_{a}H_{i}+\gamma_{a,j}+\omega_{a,i}\\ s_{i,j} & =\psi_{s}H_{i}+\gamma_{s,j}+\omega_{s,i}\\ d_{i,j} & =\psi_{d}H_{i}+\gamma_{d,j}+\omega_{d,i}\\ f_{i,j} & =\psi_{f}H_{i}+\gamma_{f,j}+\omega_{f,i}\\ v_{i,j} & =\psi_{v}H_{i}+\gamma_{v,j}+\omega_{v,i}\\ k_{i,j} & =\psi_{k}H_{i}+\gamma_{k,j}+\omega_{k,i} \end{array}$$

Here, subscripts *i* and *j* denote population and species respectively. Lower case symbols (*ϕ, a, s, d, f, v, k*) represent simulated life‐history traits that are modelled on the linear scale; that is, logit for survival and spawning duration, log for all other variables. The trait‐specific coefficients for the effect of habitat (Equation (2): *ψ*_*ϕ*_, *ψ*_*a*_, *ψ*_*s*_, *ψ*_*d*_, *ψ*_*f*_, *ψ*_*v*_, *ψ*_*k*_) were assigned from normal priors centred on zero: *N*(0,0.1). Initial model development also included replacing somatic growth with asymptotic body size (*L*_*∞*_) from the von Bertalanffy growth function, as well as maximum observed body size (*L*_max_). All populations had available data points for somatic growth rate, asymptotic body size and maximum observed body size. The Brooks–Gelman–Rubin diagnostic tool and the effective sample size of model parameters indicated poorer mixing of the MCMC in these latter models, compared to a model including somatic growth (Supporting Information Appendix S3). Consequently, all methods and results relate to the model that included growth rate.

To capture the trade‐offs between the seven life‐history traits at the between‐ and within‐species level, we modelled two random‐effect terms (*γ* and *ω* respectively) using multivariate normal distributions. The covariance structures (Σ_*i*_ and Σ_*j*_: Equation (3)) parameterizing these distributions allow the correlations, or life‐history trade‐offs, that connect the different aspects of a population’s demography to emerge during model fitting:

(3) $$\begin{array}{cc} & \left(\gamma_{i}\right)∼\text{MVN}\left(\mu_{1},\Sigma_{i}\right),\;\;\mu_{1}=\left(\mu_{1,\phi },\mu_{1,a},\mu_{1,s},\mu_{1,d},\mu_{1,f},\mu_{1,v},\mu_{1,k}\right),\;\mu_{1,*}∼N(0,15)\\ & \left(\omega_{j}\right)∼\text{MVN}\left(\mu_{2},\Sigma_{j}\right)\;,\;\;\mu_{2}=\left(\mu_{2,\phi },\mu_{2,a},\mu_{2,s},\mu_{2,d},\mu_{2,f},\mu_{2,v},\mu_{2,k}\right),\;\mu_{2,*}∼N(0,50) \end{array}$$

The mean of the multivariate normal distributions (*μ*, Equation (3)) had normal priors. Standardizing the input data to a mean of zero and a standard deviation of one meant that all traits were able to receive priors centred on zero. For the species‐level term (*γ*), being centred on zero reflects the standardized trait mean. For the population‐level term (*ω*), being centred on zero allows the population mean to operate as a classic random effect. The standard deviation in the species‐level priors was set to 0.25 (i.e. a precision of 15) to allow flexibility, and this was reduced to a standard deviation of 0.14 for the population‐level (i.e. a precision of 50). The variance–covariance matrices in the multivariate normal distributions (Σ_*i*_ and Σ_*j*_, Equation (3)) were assigned inverse Wishart prior distributions with an identity matrix for the scale matrix (Ω) and eight degrees of freedom (*df*):

(4) $$\begin{array}{cc} \Omega_{c} & =I_{n}\\ Q_{c} & ∼\text{Inv Wishart}(\Omega_{c},n+1)\\ \Sigma_{c} & =\text{Diag}\left(\xi_{c}\right)Q_{c}\text{Diag}\left(\xi_{c}\right) \end{array}$$

This equation is repeated in the model, such that *c* is either species (*i*) or population (*j*). Setting the *df* parameter for the Wishart prior to one more than the dimensions of the variance–covariance matrix (*n *= number of traits) achieves a uniform prior distribution on the individual correlation parameters; that is, an equally likely probability between −1 and 1 (Gelman & Hill, 2006). A scaling parameter (*ξ*) assigned from a uniform prior: *U*(0,5), was also incorporated to overcome constraints on the scale parameters associated with an inverse‐Wishart model (Gelman & Hill, 2006).

Observed life‐history data for each trait (*Φ, A, S, D, F, V, K*) were incorporated through an observation component that reflected a combination of measurement error and process variability in the data (e.g. spatial and temporal variation). Upper case notation is used to denote observed, population‐specific data. Data were included at the study level (*p*) for each species (*i*) and population (*j*) :

(5) $$\begin{array}{cc} & \text{logit}\left({\Phi_{p,}}_{i,j}\right)∼N\left(\phi_{i,j},\tau_{\phi }\right)\\ & \text{log}\left({A_{p,}}_{i,j}\right)∼N\left(a_{i,j},\tau_{a}\right)\\ & \text{log}\left({S_{p,}}_{i,j}\right)∼N\left(s_{i,j},\tau_{s}\right)\\ & \text{logit}\left({D_{p,}}_{i,j}\right)∼N\left(d_{i,j},\tau_{d}\right)\\ & \text{log}\left({F_{p,}}_{i,j}\right)∼N\left(f_{i,j},\tau_{f}\right)\\ & \log\left({V_{p,}}_{i,j}\right)∼N\left(v_{i,j},\tau_{v}\right)\\ & \text{log}\left({K_{p,}}_{i,j}\right)∼N\left(k_{i,j},\tau_{k}\right) \end{array}$$

Variance was assumed to be Gaussian, where the reconstructed population values (*ϕ*_*i*,*j*_, *a*_*i*,*j*_, *s*_*i*,*j*_, *d*_*i*,*j*_, *f*_*i*,*j*_, *v*_*i*,*j*_, *k*_*i*,*j*_) were taken from Equation (2), and the precision (*τ*_ϕ_, *τ*_a_, *τ*_s_, *τ*_d_, *τ*_f_, *τ*_v_, *τ*_k_) was set from a normal distribution centred on 20: *N*(20,0.1). This assumes a reasonably high precision on the reconstructed value for data‐limited traits; however, for data‐rich traits, this prior distribution will allow the precision to travel into the tails, that is, become high or low in line with the observed variance in the data. Thus, in addition to imputing missing values, posterior distributions for the observed data points were also obtained. This acknowledges that even observed life‐history data are a sample from a theoretical population of possible trait‐specific values whose mean and credible intervals can be obtained. Although this exercise is similar to the traditional approach of sampling distributions, the joined inference across all data prevents the resulting parameter posteriors from being restricted by small sample sizes.

### 2.3 Model validation

We cross‐validated the predictive capacity of the life‐history model by examining whether the model was able to accurately predict the observed life‐history from a single, readily available trait: somatic growth. Model performance was examined under two scenarios: (a) when other populations of that species have life‐history data, and (b) when the species is represented by a single population. For the first scenario, we removed the life‐history data for a population with data for all seven life‐history traits, retaining somatic growth rate only; here, we chose the southern Pacific population of albacore tuna. This species is represented by five additional populations in the model (Figure 1; Supporting Information Table S1). For the second scenario, we removed the life‐history data for a species represented by a single population, retaining somatic growth rate only; here, we chose Southern bluefin tuna (Figure 1; Supporting Information Table S1). We also ran an additional six model simulations to examine how the predicted life‐history of Southern bluefin tuna changed with the addition of each life‐history trait, alongside growth rate.

The significance of the habitat term in the demographic functions was examined based on the posterior credible interval of the coefficient parameters (Equation (2): *ψϕ*,* ψ*_*a*_, *ψ*_*s*_, *ψ*_*d*_, *ψ*_*f*_, *ψ*_*v*_, *ψ*_*k*_), as well as a comparison of the imputed traits and model fit obtained from the full model versus one where insignificant habitat terms were removed. Parameters where the 97.5 posterior credible interval spanned zero were deemed insignificant. For many species, especially fish, information on fecundity may not be known. Therefore, we also examined the dependence of the model on the inclusion of the four fecundity metrics by running the model with a reduced set of traits: survival, age of maturity and somatic growth. We compared the imputed traits and associated credible intervals from this model with the output from the full model that also included metrics of fecundity.

## 3 RESULTS

### 3.1 Hierarchical modelling of life‐history trade‐offs

**Figure 2:**
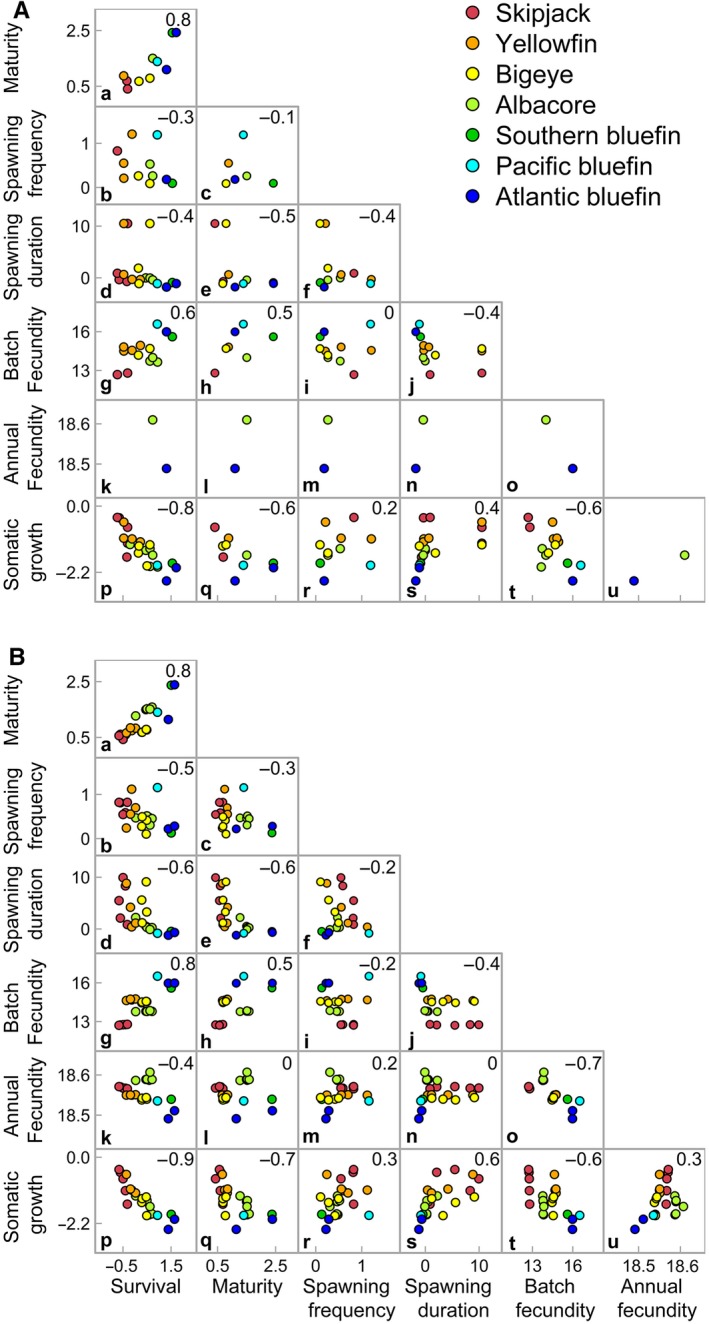
The strength of trade‐offs between a seven trait life‐history strategy for the seven species of principal market tunas. (A) Original data (based on mean values) and (B) reconstructed data (median posterior values), both on the scale of the linear predictor. Species are demarked using colour. Colour ramp corresponds to the observed gradient in the observed data for somatic growth rate (Juan‐Jordá et al., 2016), from fast to slow, within the tropical and temperate groupings. Pearson correlation coefficients between traits shown in the top left hand corner of each panel, not shown for the original dataset of annual fecundity due to sample size. Units: age of maturity (years), spawning frequency (days), spawning duration (proportion of year), batch fecundity (number of oocytes per individual per batch), annual fecundity (number of ooctyes)

**Figure 3:**
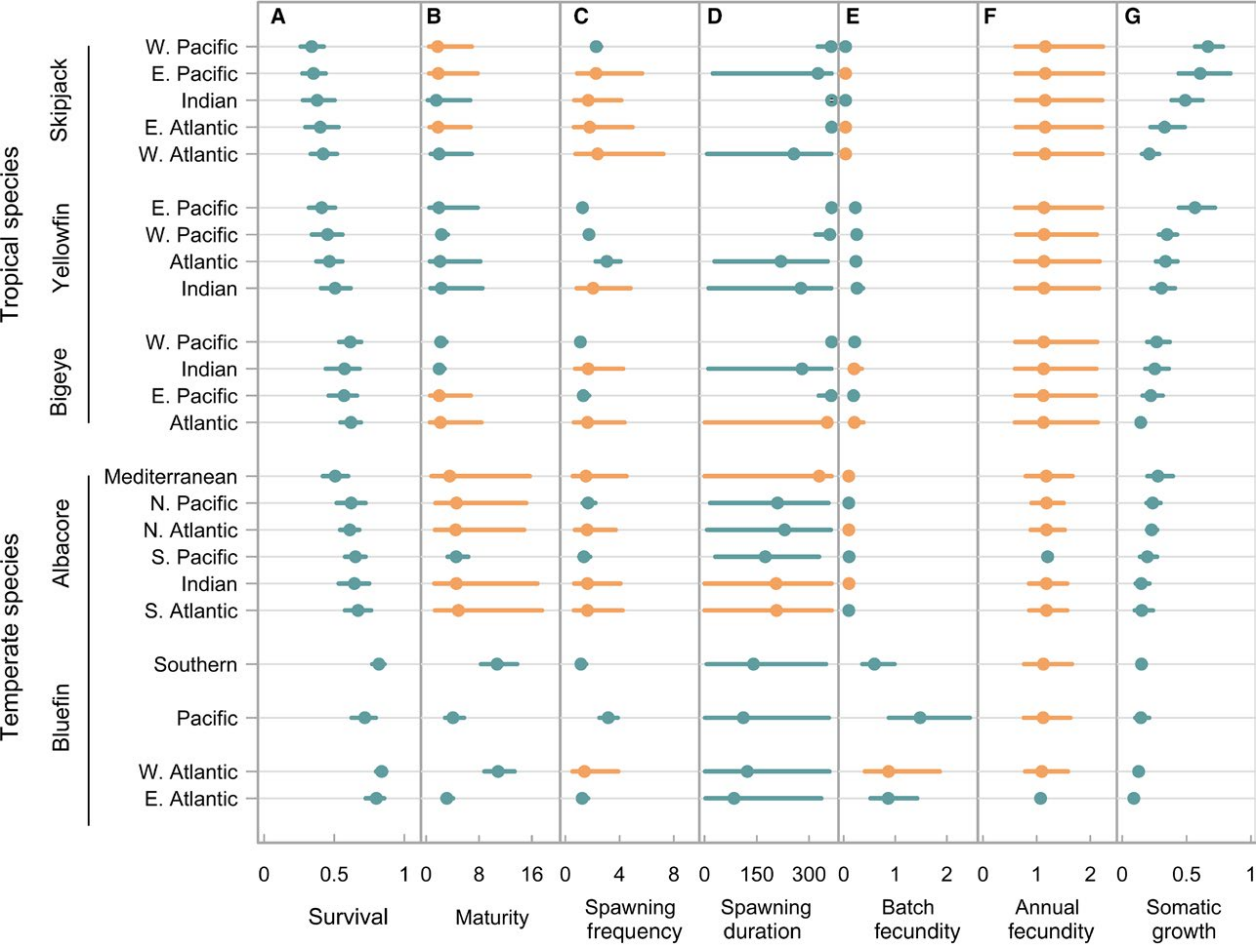
Reconstructed life‐history traits by habitat, species and population. Median values are shown as points and 95% credible intervals are shown as bars. Blue indicates parameters with data available (all observed data points fell within the credible intervals), orange indicates parameters with data missing in the original dataset. Species and populations listed in the observed order of somatic growth rate (Juan‐Jordá et al., 2016), from fast to slow, within the tropical and temperate groupings. Somatic growth is adjusted to include population‐specific residuals. Units: age of maturity (years), spawning frequency and spawning duration (days), batch fecundity (number of oocytes per individual per batch, ×10^7^), annual fecundity (number of ooctyes, ×10^8^)

The vectors of predicted, or reconstructed, life‐history traits (*ϕ,a,s,d,f,v,k*, Equation (2)) are presented in Figures 2 and 3. Pearson correlation coefficients between traits were estimated using median posterior values. Correlations between life‐history traits became, on average, 14% stronger following reconstruction (Figure 2), and all original data points remained within the 95% credible intervals of the reconstructed values. Correlations between all traits and survival, age of maturity and somatic growth were consistently strong (−0.3 ≥ *ρ* ≥ 0.3, Figure 2B), with the exception of age of maturity and annual fecundity (*ρ* = 0, Figure 2B–I). Correlations between the four fecundity traits were predominantly weaker (−0.2 ≤ *ρ* ≤* *0.2, Figure 2B–f,i,m,n), with the exception of annual fecundity and batch fecundity (*ρ* = −0.7, Figure 2B–o), and batch fecundity and spawning duration (*ρ* = −0.4, Figure 2B–j).

The reconstructed life‐history strategies (Supporting Information Table S3) of the principal market tunas appeared along a thermal‐growth gradient, whereby tropical species had faster rates of somatic growth compared to species that spend a large portion of their annual cycle in temperate waters (Figure 2). In addition, there was a gradual increase in survival rates from the fast‐growing tropical species to the slower growing temperate species (Figure 3A). The tropical species also matured earlier compared to the temperate species and returned predominantly larger credible intervals for annual fecundity (Figure 3B and F). The relationship with maturation was further demonstrated by the coefficient of the relationship with biome (97.5 credible interval for ψ_a_, Equation (2): −2.000 to −0.003: Figure 3B). Based on the median values, spawning duration was longer in tropical species (Figure 3D). In contrast, spawning frequency and batch fecundity did not clearly differ between the two habitat groupings (Figure 3C and E), although batch fecundity increased and was more variable for the three slow‐growing bluefin species (Figure 3E).

Large intra‐population variation in the raw data for spawning duration was not reduced through model fitting (see Supporting Information Figure S1). Furthermore, the large disparity between the mean published values of maturation for the two populations of Atlantic bluefin tuna was maintained (Figure 3B, Supporting Information Figure S1). Intra‐species variability appeared as different median estimates of traits within species, for example, in survival (Figure 3A) and somatic growth (Figure 3G), as well as differences in the credible interval for each trait, for example, in age of maturity (Figure 3B), spawning duration (Figure 3D), spawning frequency (Figure 3C) and annual fecundity (Figure 3F; Supporting Information Table S3). Within the three bluefin tuna species, the breeding strategy of Pacific bluefin tuna appeared more distinct, compared to the other two species (Figure 3).

### 3.2 Validation testing

**Figure 4:**
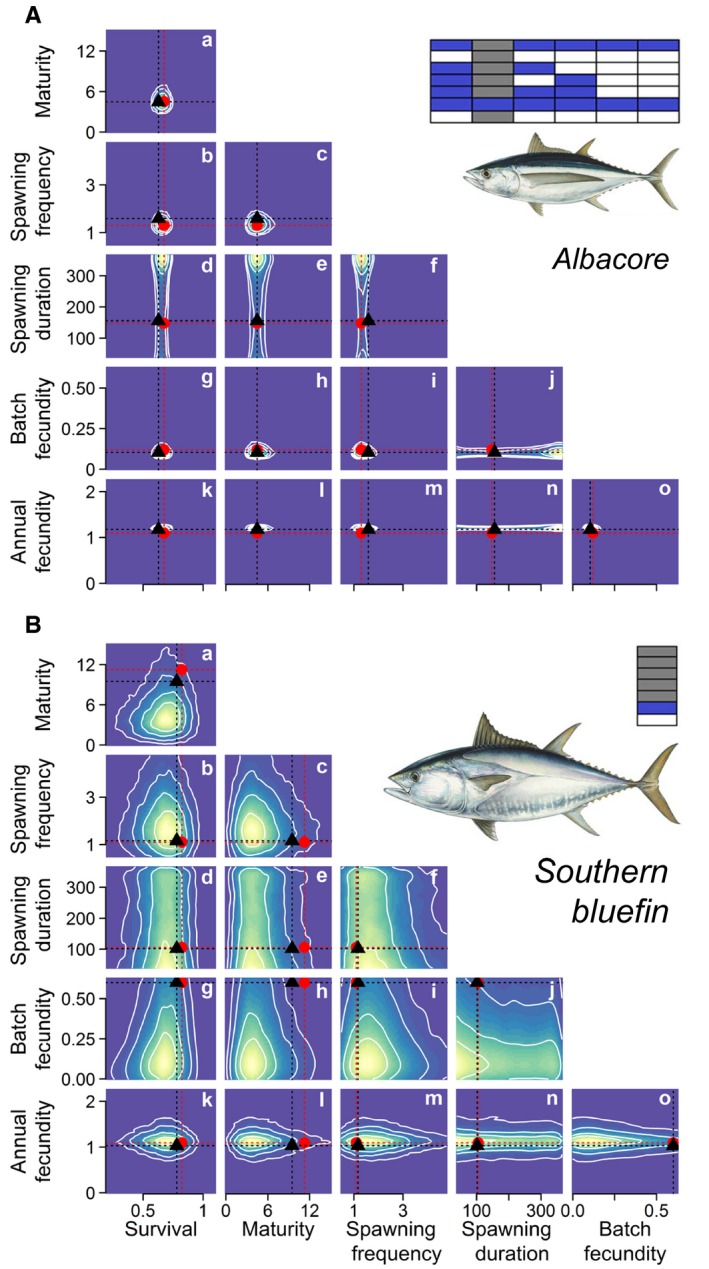
Predicting the life‐history information for (A) the Southern Pacific population of albacore tuna, (B) Southern bluefin tuna. Two‐dimensional kernel density plots correlate the full posterior distribution for each life‐history trait combination. Colour ramp corresponds to the probability associated with values: highest probability in yellow and lowest probability in blue. Original data points are shown as red circles and red dashed lines, and the mean imputed values from the model including all available data are shown as black triangles and black dashed lines. Data availability for each trait are shown as stacks of blocks (see Figure 1); shaded blocks indicate that life‐history data were available for that population, those shaded grey represent data removed and imputed in the validation test. Tuna pictures from Diane Rome Peebles. See Figure 3 for trait units

Life‐history traits were recreated convincingly for a population that was represented by multiple other populations of the same species (south Pacific albacore tuna), with the exception of spawning duration (Figure 4A). For all other traits, median posterior values were similar to the original data as well as the values reconstructed by the model that included all available data. Furthermore, traits were returned within tight credible intervals. For a species that was represented by a single population (Southern bluefin tuna), the model convincingly recreated the median values for survival, spawning frequency and annual fecundity (Figure 4B). However, maturity was imputed to be lower than the original data and the median value reconstructed by a model that incorporated all available data. Furthermore, the credible intervals of reconstructed traits increased considerably, compared to the model including all available data (Supporting Information Table S4). Inference was only improved by refitting the model with the respective life‐history data incorporated (Supporting Information Figure S2).

Examination of the coefficient parameters for the effect of habitat preference demonstrated that age of maturity was the only trait with a step difference between biomes, as opposed to a more continual change from the fastest growing tropical species to the slowest growing temperate species. Removing the remaining habitat terms from the demographic functions generated marginal changes to the posterior credible intervals for annual fecundity and the median values for spawning duration. Furthermore, the remaining traits were largely unchanged (Supporting Information Figure S3). However, the overall fit of a model without a complete set of habitat terms was reduced compared to a model including these terms (minimum effective sample for model without habitat terms = 2,604, all $\hat{r}$ ≤ 1.13; minimum effective sample size for model including habitat terms = 3,764, all $\hat{r}$ ≤ 1.01). Removing the fecundity traits from the inference framework resulted in a marginal change in the credible intervals of the reconstructed life‐history parameters (less than 5% for the majority of populations, max. 7%; Supporting Information Table S5). The imputed median life‐history traits were also highly correlated with those estimated by the model that included these additional four metrics of fecundity (Supporting Information Figure S4).

## 4 DISCUSSION

In this study, we applied a formal hierarchical Bayesian approach that connects different life‐history traits in order to quantify trade‐offs and synergies (i.e. negative and positive correlations) and predict missing values. This approach provides potentially more precise estimates of life‐history traits for use in population assessments, compared to surrogate data assigned from similar species or other populations of the same species. We compiled our starting dataset from an extensive repository of life‐history studies on scombrids (Juan‐Jordá et al., 2016) that offers a more exhaustive coverage of available data sources than other global sources of life‐history parameters, such as FishBase. We apply the approach to the 23 populations of principal market tuna recognized globally. As these are closely related species that inhabit large geographic areas and migrate over broad latitudinal gradients, a straightforward assignment of missing life‐history information using phylogenetic or environmental determinants (e.g. Thorson et al., 2017) is insufficient to resolve life‐history differences among populations. The seven‐trait life‐history strategies reconstructed here provide insights into the variation within‐ and between‐species. By taking some degree of observation error and spatio‐temporal variation into account during model fitting, we also quantify the uncertainty associated with each reconstructed value. Propagating this uncertainty into projections of population change allows realistic estimates of extinction and over‐exploitation risk to be quantified.

Our framework performed well at reconstructing missing life‐history values when information was available for other populations of the same species. Furthermore, inference of survival and age of maturity were not degraded by running the model without the four fecundity metrics that are often more difficult to measure in batch spawning income breeders, like tunas. The marginal change in model fit created by adding (or removing) the fecundity metrics is likely to reflect the large gaps in these datasets, such that the other life‐history traits have a stronger influence on resolving fecundity than vice versa. Inference was weaker when predicting the life‐history traits of a single‐population species (Southern bluefin tuna). For this species, the median posterior values were relatively accurate for traits where the original data appeared close to the inter‐species linear regression with growth rate (e.g. survival and annual fecundity). However, the observed values of maturation and batch fecundity are both higher than expected for its growth rate, that is, these values have large residuals from the linear regression, thus reducing predictive capacity (Figure 3B and E). Furthermore, Southern bluefin tuna exists at the edge of the parameter space where the likelihood surface may be less well defined. This is likely to have contributed to the wider credible intervals resulting from this aspect of the model validation exercise.

The direction and strength of the evolutionary correlations that constrain the life‐history strategies emerged during model fitting. In agreement with theory, several key life‐history traits (somatic growth, survival and maturity) appeared along a gradient. Here, a slow life‐history strategy was characterized by slower rates of body growth, higher rates of adult survival and higher ages of maturity, while a fast life‐history strategy included faster rates of body growth, lower rates of survival and earlier maturation (Figure 2; Stearns, 1992). Previously undefined life‐history trade‐offs between the different aspects of reproduction for batch‐spawning income breeders were also inferred through model fitting. In general, a slow life‐history strategy was associated with shorter spawning durations and higher batch fecundities, while a fast life‐history strategy had longer spawning durations and lower batch fecundities. Colder habitats select for slower life histories in fishes, including shorter spawning seasons, because offspring survival is strongly linked to seasonal food availability, for example spring algal blooms (Lowerre‐Barbieri, Ganias, Saborido‐Rey, Murua, & Hunter, 2011). However, the described trade‐offs between reproductive traits may also imply some directional selection between spawning duration and batch fecundities, whereby temperate species can produce large batches of oocytes because the energetic cost is temporally restricted. Variable strategies within the slow‐growing bluefin tunas also indicate population‐ or species‐specific trade‐offs. For example, Pacific bluefin tuna returned elevated median values for batch fecundity and spawning frequency and lower values for survival and age of maturity, relative to the other bluefin species. This result suggests that it is not possible for these large, slow‐growing tunas to increase their reproductive output without trading‐off their survival rates.

Modelling the full set of life‐history traits across a species’ range allows differences between populations to be examined. Clear differences among populations, as well as species, were observed in somatic growth, spawning frequency and spawning duration (Figure 3C, D, G) that may reflect diverging strategies associated with habitat or lifestyle. In agreement with Juan‐Jordá et al. (2013, 2015), broad similarities in survival, maturation and spawning duration could be drawn based on whether the species predominantly inhabits tropical or temperate waters during its annual cycle. The slower life‐history strategies of temperate species, especially bluefin tunas, is likely to be a contributing factor in their overexploitation (Juan‐Jordá et al., 2015). Tropical species also returned predominantly larger credible intervals associated with annual fecundity, compared to the temperate species. However, the median values appeared to be similar across the two habitat groupings, despite temperate species spawning for a shorter period compared to tropical species. The smallest credible intervals for annual fecundity were obtained for the two temperate populations with observations for this trait. Therefore, this apparent trend is likely to reflect data availability, such that more empirical estimates will improve parameter estimation of annual fecundity, as well as the resolution of associated traits and trade‐offs. Complex breeding strategies mean that estimates of annual fecundity are particularly difficult to obtain for these species (Farley, Williams, Hoyle, Davies, & Nicol, 2013). The imputed credible intervals thus provide a unique insight into breeding strategies.

By reconstructing missing trait values based on the life‐history correlations that connect different characteristics, we imputed values that are potentially more precise single estimates than the measured data. This is likely to be the case for traits that are difficult to resolve or that demonstrate large intra‐population variability, such as batch fecundity and spawning duration, respectively. However, for traits that are reliably resolved, such as growth rate, there is an argument for not using reconstructed values. The life‐history trait values reconstructed in this study largely reflect the population‐level mean value for the available data, indicating that shrinkage towards the species‐level mean during model fitting is limited (Supporting Information Figure S1). By limiting the number of covariates in the demographic functions (Equation (2)), model fitting is predominantly driven by the original data and the variance–covariance matrix connecting traits. Thus, divergent traits within species were maintained, for example age of maturity for the two populations of Atlantic bluefin tuna. There is a current debate about this reported divergence (ICCAT, 2017). The previously published age of maturity for the eastern population may be an underestimate because samples outside the Mediterranean are not included in the original analyses, increasing the proportion of mature fish from the resident population (ICCAT, 2014). Thus, the inclusion of future data as model input may change the inference of this trait.

The described approach for imputing missing life‐history data could potentially be applied to any data‐limited group of species. Within a fisheries context, the reconstructed life‐history data can be employed in data‐limited assessments, such as ecological risk assessments and length‐based models (Hordyk, Ono, Prince, & Walters, 2016; Rudd & Thorson, 2018). In addition, the approach has immediate application to the world‐wide management of fisheries for tuna in which the steepness of the Stock Recruitment Relationship (SRR) is used. We illustrate the ideas for the Beverton‐Holt SRR (BH‐SRR), but our results apply to other SRRs as well. With the BH‐SRR, we predict the number of recruits, that is, individuals added to the population in the youngest age class (*R*), as a function of the spawning biomass (*B*_*s*_), which we write as $R\left(B_{s}\right)=\frac{\lambda B_{s}}{1+\beta B_{s}}$ where *λ* and *β* are parameters. The former is a measure of the maximum per capita productivity, that is, related to annual fecundity, and the latter a measure of the strength of density dependence. Steepness (*h*) is defined to be the reproduction when spawning biomass is 20% of its unfished level (*B*_0_) relative to reproduction at the unfished level; $h=\frac{R(0.2B_{0})}{R(B_{0})}$ (see Mangel et al., 2010, 2013 for reviews). For the BH‐SRR, steepness is (Mangel et al., 2010, 2013):

(6) $$h=\frac{\lambda \overset{¯}{W}}{4+\lambda \overset{¯}{W}}$$

where $\overset{¯}{W}$ is the average spawning biomass of a female that has survived to age *a* (*Φ*(*a*)), and the probability of being mature at age *a* (*p*_*m*_(*a*)) and mass at age *a* (*W*(*a*)) according to $\overset{¯}{W}=\sum_{a}\Phi \left(a\right)p_{m}\left(a\right)W\left(a\right)$. Our methodology provides values for survival and maturation and can easily be adapted to include mass as an additional life‐history trait.

Application of Equation (6) to determine steepness requires knowing *λ*, which is the product of mass‐specific fecundity δ (eggs, or larvae for those species with live birth, per unit spawning biomass) and survival from the egg or larval stage until recruitment to the population *ϕ*_EL_, thus, *λ* = *δϕ*_EL_. Survival to recruitment into the population can be modelled using well‐established allometries between size and mortality (e.g. Brodziak, Mangel, & Sun, 2015; Kai & Fujinami, 2018; Mangel, Brodziak, & DiNardo, 2010; Simon et al., 2012), and the described hierarchical model of life‐history traits allows the computation of total fecundity. In the Supporting Information Appendix S4, we show how to obtain mass‐specific fecundity from total fecundity under appropriate assumptions. Thus, once mass‐specific fecundity is known, the parameter *λ* can be determined for a data‐poor stock.

Our analyses reveal that by considering multiple life‐history traits, populations and species simultaneously in a Bayesian framework, it is possible to statistically maximize the utility of sparse datasets, quantify the trade‐offs that connect different aspects of an organism's life‐history and access traits that are difficult to measure empirically. The percentage of missing life‐history data will inevitably be higher for other, commercially less important groups of fishes, such that a Bayesian hierarchical approach informed by the principles of life‐history evolution, including metabolic theory, could further assist in population‐specific trait reconstruction.

## AUTHORS’ CONTRIBUTIONS

All authors contributed to the design and planning of this study; M.J.J.J. collated the scombrid life‐history dataset; C.H. conducted all analyses and wrote the first draft of the manuscript, and all authors contributed substantially to revisions and gave final approval for publication.

## Data Availability

The tuna dataset was compiled from https://doi.org/10.1890/15-1301.1 (Juan‐Jordá et al., 2016), supplemented with individual values from https://doi.org/10.1016/j.seares.2012.08.005 (Aranda, Medina, Santos, Abascal, & Galaz, 2013); https://doi.org/10.1371/journal.pone.0060577 (Farley et al., 2013); https://www.iccat.int/Documents/CVSP/CV068_2012/n_2/CV068020387.pdf (ICCAT 2012); https://doi.org/10.1111/j.1095-8649.2002.tb02398.x (Medina, Abascal, Megina, & Garcia, 2002) and https://doi.org/10.7755/fb.111.3.4 (Zudaire, Murua, Grande, & Bodin, 2013). The data read‐in file for the life‐history model are available in Supporting Information Appendix S2.
