# Supplementary material for: Global reconstruction of life‐history strategies: A case study using tunas
Source: J Appl Ecol. 2019 Feb 1;56(4):855–65. doi: 10.1111/1365-2664.13327 (PMC6559282; doi:10.1111/1365-2664.13327)
Supplement: Supplementary file 1 [file JPE-56-855-s001.docx]

**Supporting information for Horswill et al. *Global reconstruction of life-history strategies***


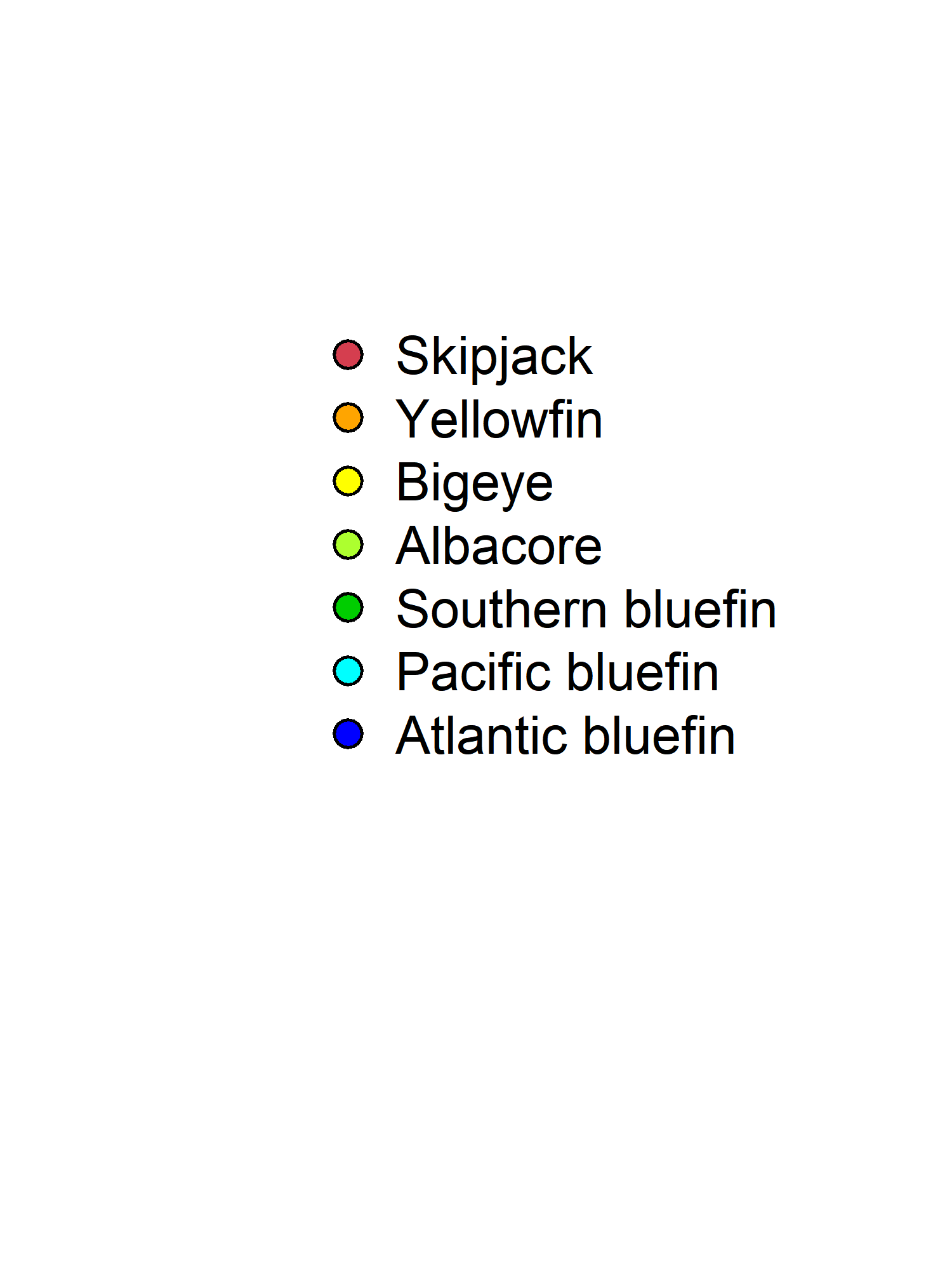

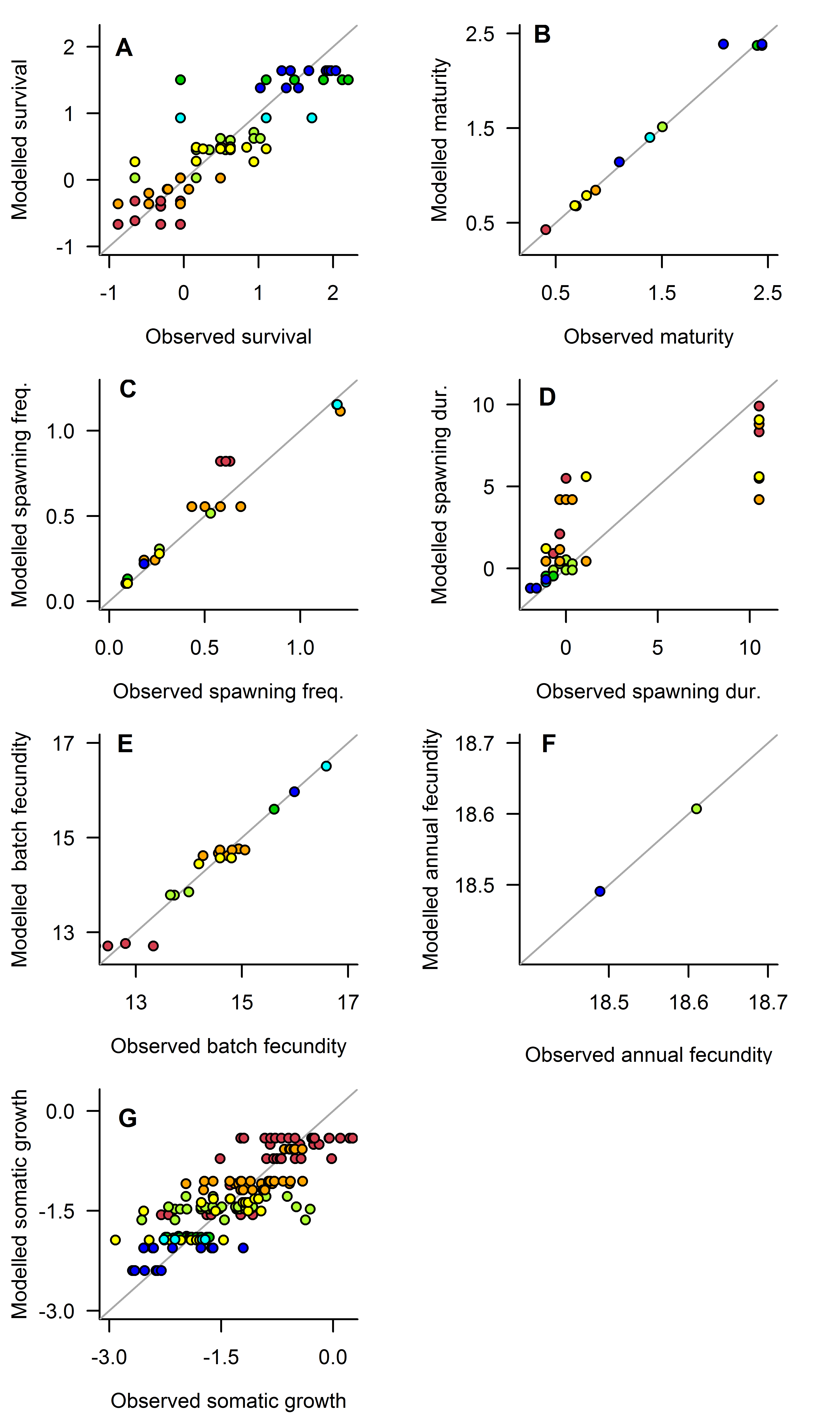


Figure S1. Correlation of raw (observed) life-history data and reconstructed (modelled) median values for each trait. Species are demarked using colour. Colour ramp corresponds to the observed gradient in the observed data for somatic growth rate, from fast to slow, within the habitat groupings: tropical and temperate. All values are on the scale of the linear predictor, 1:1 line shown as grey line for reference.
