# Supplementary material for: Global reconstruction of life‐history strategies: A case study using tunas
Source: J Appl Ecol. 2019 Feb 1;56(4):855–65. doi: 10.1111/1365-2664.13327 (PMC6559282; doi:10.1111/1365-2664.13327)
Supplement: Supplementary file 2 [file JPE-56-855-s002.docx]

**Supporting information for Horswill et al. *Global reconstruction of life-history strategies***

Figure S2. Refitting the life-history information for the Southern bluefin tuna population. The six simulations represent the life history information supplied as data: A. Somatic growth only, B. Somatic growth and survival, C. Somatic growth and maturity, D. Somatic growth and spawning frequency, E. Somatic growth and spawning duration, F. Somatic growth and batch fecundity. Two-dimensional kernel density plots between the posterior values of each life-history trait. Original data points are shown as red circles and red dashed lines, and the mean imputed values from the full model are shown as black triangles and black dashed lines.
