# Supplementary material for: Global reconstruction of life‐history strategies: A case study using tunas
Source: J Appl Ecol. 2019 Feb 1;56(4):855–65. doi: 10.1111/1365-2664.13327 (PMC6559282; doi:10.1111/1365-2664.13327)
Supplement: Supplementary file 3 [file JPE-56-855-s003.docx]

**Supporting information for Horswill et al. *Global reconstruction of life-history strategies***


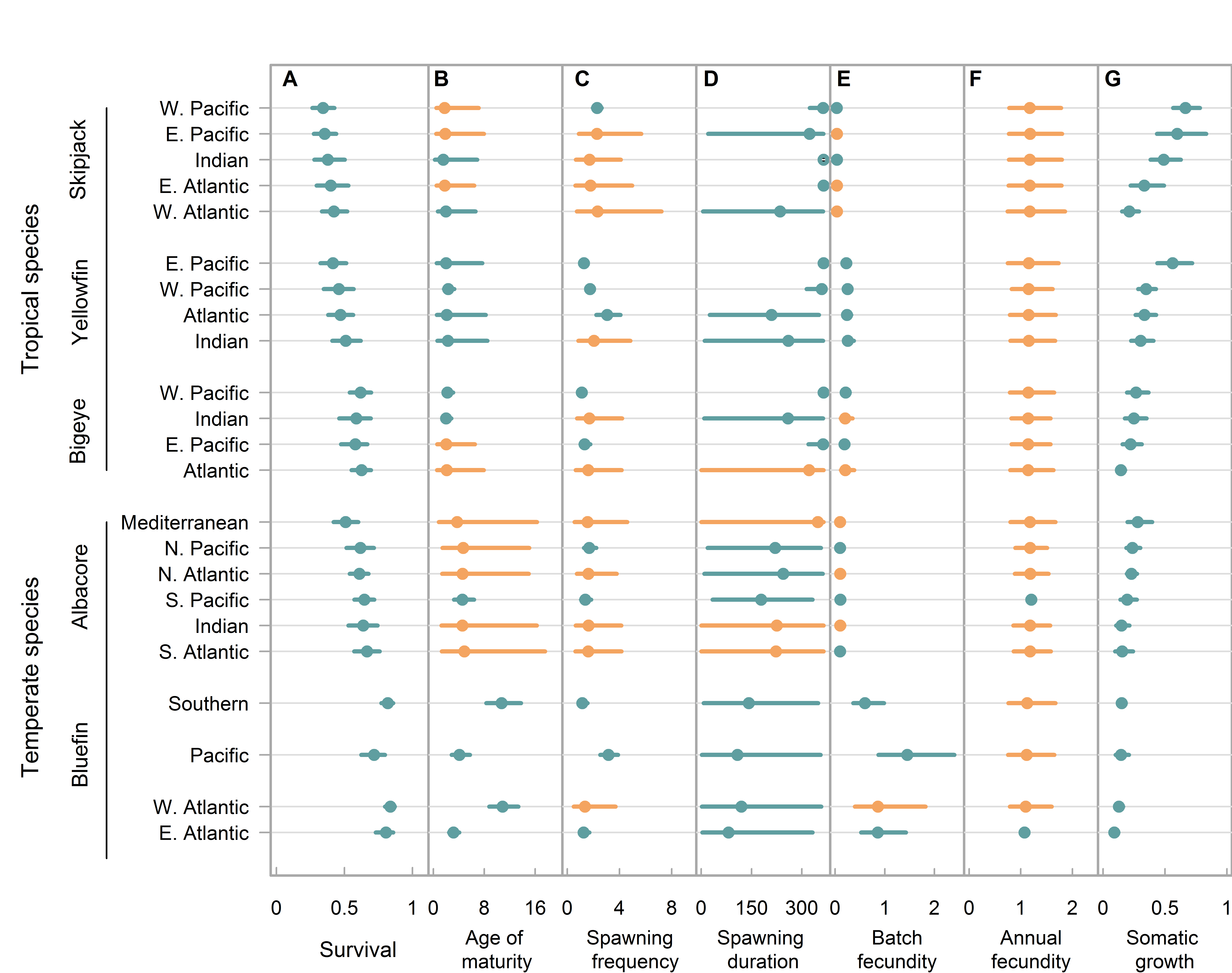


Figure S3. Reconstructed life-history traits by habitat, species and population for a model without the habitat covariate included in all demographic functions, with the exception of maturity. The 95% credible intervals are shown as bars. Blue indicates parameters that also had data available (all observed data points fell the credible intervals), orange indicates parameters with data missing from the original dataset. Species and populations listed in the observed order of somatic growth rate, from fast to slow, within the tropical and temperate groupings. Somatic growth is adjusted to include population specific residuals. Units: age of maturity (years), spawning frequency and spawning duration (days), batch fecundity (number of oocytes per individual per batch, x10^7^), annual fecundity (number of ooctyes per individual per year, x10^8^).
