# Supplementary material for: Global reconstruction of life‐history strategies: A case study using tunas
Source: J Appl Ecol. 2019 Feb 1;56(4):855–65. doi: 10.1111/1365-2664.13327 (PMC6559282; doi:10.1111/1365-2664.13327)
Supplement: Supplementary file 4 [file JPE-56-855-s004.docx]

**Supporting information for Horswill et al. *Global reconstruction of life-history strategies***


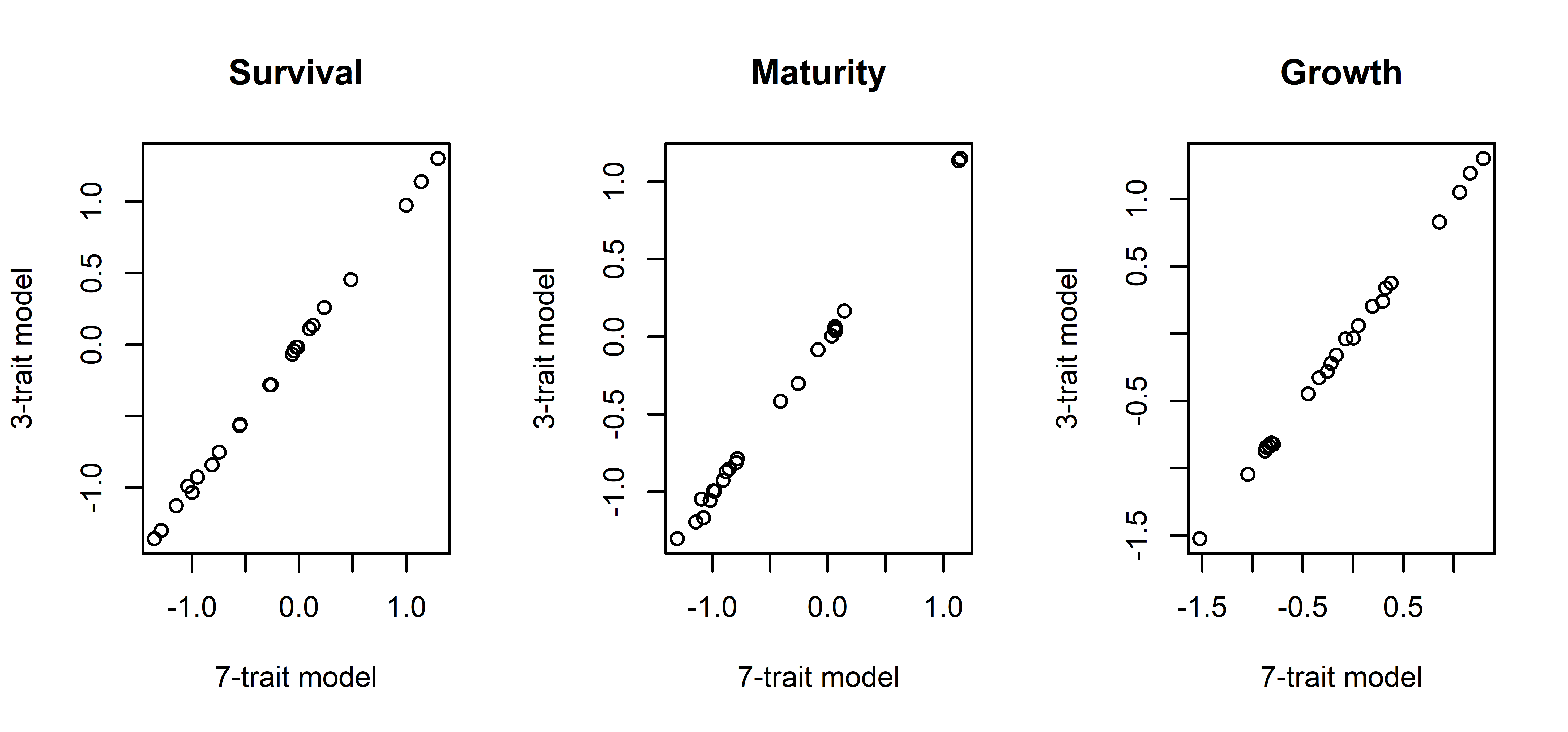


Figure S4. Correlation of the median posterior values for survival, age of maturity and somatic growth between a model including all seven life-history traits and a model including three traits only (survival, age of maturity and growth).
