# Supplementary material for: Global reconstruction of life‐history strategies: A case study using tunas
Source: J Appl Ecol. 2019 Feb 1;56(4):855–65. doi: 10.1111/1365-2664.13327 (PMC6559282; doi:10.1111/1365-2664.13327)
Supplement: Supplementary file 5 [file JPE-56-855-s005.docx]

**Supporting information for Horswill et al. *Global reconstruction of life-history strategies***

Table S1. Number of studies available for each life-history trait across the 23 populations for principal market tuna.

| Species | Population | Somatic growth rate | Asymptotic body size | Maximum body size | Maximum age (used to derive rates of survival) | Age maturity | Spawning frequency | Spawning duration | Batch fecundity | Annual Fecundity |
| --- | --- | --- | --- | --- | --- | --- | --- | --- | --- | --- |
| Skipjack tuna *Katsuwonus pelamis* | Eastern Atlantic population | 3 | 3 | 5 | 1 | - | - | 1 | - | - |
|  | Eastern Pacific population | 5 | 5 | 4 | 3 | - | - | 1 | - | - |
|  | Indian population | 9 | 9 | 18 | 1 | 1 | - | 5 | 1 | - |
|  | Western Atlantic population | 6 | 6 | 9 | 3 | 1 | - | 1 | - | - |
|  | Western Pacific population | 22 | 22 | 25 | 3 | - | 4 | 2 | 3 | - |
| Albacore tuna *Thunnus alalunga* | Indian population | 4 | 4 | 7 | 1 | - | - | - | - | - |
|  | Mediterranean population | 4 | 4 | 5 | 5 | - | - | - | - | - |
|  | Northern Atlantic population | 18 | 18 | 17 | 5 | - | - | 1 | - | - |
|  | Northern Pacific population | 10 | 10 | 12 | 1 | - | 1 | 2 | 1 | - |
|  | Southern Atlantic population | 2 | 2 | 4 | 2 | - | - | - | 1* | - |
|  | Southern Pacific population | 4 | 4 | 4 | 4 | 1 | 1 | 5 | 1 | 1* |
| Yellowfin tuna *Thunnus albacores* | Atlantic population | 9 | 9 | 8 | 3 | - | 1 | 3 | 1 | - |
|  | Eastern Pacific population | 9 | 9 | 10 | 3 | - | 2 | 1 | 2 | - |
|  | Indian population | 6 | 6 | 11 | 2 | - | - | 1 | 1* | - |
|  | Western Pacific population | 13 | 13 | 17 | 1 | - | 4 | 5 | 3 | - |
| Southern bluefin tuna *Thunnus maccoyii* | Southern population | 14 | 14 | 10 | 7 | 2 | 1 | 2 | 1 | - |
| Bigeye tuna *Thunnus obesus* | Atlantic population | 12 | 12 | 13 | 5 | - | - | - | - | - |
|  | Eastern Pacific population | 4 | 4 | 6 | 2 | - | 1 | 2 | 1 | - |
|  | Indian population | 4 | 4 | 5 | 1 | 1 | - | 1 | - | - |
|  | Western Pacific population | 5 | 5 | 12 | 4 | 1 | 2 | 2 | 2 | - |
| Pacific bluefin tuna *Thunnus orientalis* | Pacific population | 4 | 4 | 7 | 3 | 1 | 1 | 1 | 1 | - |
| Atlantic bluefin tuna *Thunnus thynnus* | Eastern Atlantic population | 6 | 6 | 10 | 3 | 2 | 1 | 2 | 1* | 1* |
|  | Western Atlantic population | 10 | 10 | 14 | 8 | 3 | - | 1 | - | - |

*Supplementary studies to Juan-Jordá et al. (2016):

Aranda, G., A. Medina, A. Santos, F. J. Abascal, and T. Galaz. 2013. Evaluation of Atlantic bluefin tuna reproductive potential in the western Mediterranean Sea. Journal of Sea Research 76:154–160.

Farley, J. H., A. J. Williams, S. D. Hoyle, C. R. Davies, and S. J. Nicol. 2013. Reproductive dynamics and potential annual fecundity of South Pacific albacore tuna (*Thunnus alalunga*). PLoS ONE 8:e60577.

ICCAT. 2012. Report of the 2011 ICCAT South Atlantic and Mediterranean albacore stock assessment sessions. Col. Vol. Sci. Pap. ICCAT 68:387–497.

Medina, A., F. J. Abascal, C. Megina, and A. Garcia. 2002. Stereological assessment of the reproductive status of female Atlantic northern bluefin tuna during migration to Mediterranean spawning grounds through the Strait of Gibraltar. Journal of Fish Biology 60:203–217.

Zudaire, I., H. Murua, M. Grande, and N. Bodin. 2013. Reproductive potential of Yellowfin Tuna (*Thunnus albacares*) in the western Indian ocean. Fishery Bulletin 111:252–264.
