# Supplementary material for: Global reconstruction of life‐history strategies: A case study using tunas
Source: J Appl Ecol. 2019 Feb 1;56(4):855–65. doi: 10.1111/1365-2664.13327 (PMC6559282; doi:10.1111/1365-2664.13327)
Supplement: Supplementary file 6 [file JPE-56-855-s006.docx]

**Supporting information for Horswill et al. *Global reconstruction of life-history strategies***

Table S2. Key parameters in the model of life-history traits and method of estimation

| Parameter | Parameter notation | Equation | Estimation | Prior |
| --- | --- | --- | --- | --- |
| Coefficient for habitat term | *$\psi$* | 2 | Estimated | $N(0, 5)$ |
| Species-specific mean trait value | *$\gamma$* | 2 | Estimated | $MVN(\mu_{1},\Sigma_{i})$ |
| Population-specific trait values | *$\omega$* | 2 | Estimated | $MVN(\mu_{2},\Sigma_{j})$ |
| Mean expectation for species-level MVN | $\mu_{1}$ | 3 | Estimated | $N(0, 15)$ |
| Mean expectation for population-level MVN | $\mu_{2}$ | 3 | Estimated | $N(0, 50)$ |
| Variance-covaraince matrix for species-level MVN | $\Sigma_{i}$ | 3 | Estimated | $InvWishart(\Omega_{i},8)$ |
| Variance-covaraince matrix for population-level MVN | $\Sigma_{j}$ | 3 | Estimated | $InvWishart(\Omega_{j},8)$ |
| Scale matrix for species-level Inverse Wishart | $\Omega_{i}$ | 4 | Fixed | $I_{8}$ |
| Scale matrix for population-level Inverse Wishart | $\Omega_{j}$ | 4 | Fixed | $I_{8}$ |
| Scaling parameter for species-level scale matrix | $\xi_{i}$ | 4 | Estimated | $U(0,5)$ |
| Scaling parameter for population-level Inverse Wishart | $\xi_{j}$ | 4 | Estimated | $U(0,5)$ |
| Precision of observation component | $\tau$ | 5 | Estimated | $N(20, 0.1)$ |
