# Supplementary material for: Global reconstruction of life‐history strategies: A case study using tunas
Source: J Appl Ecol. 2019 Feb 1;56(4):855–65. doi: 10.1111/1365-2664.13327 (PMC6559282; doi:10.1111/1365-2664.13327)
Supplement: Supplementary file 7 [file JPE-56-855-s007.docx]

**Supporting information for Horswill et al. *Global reconstruction of life-history strategies***

ST3. Imputed life-history traits for each population of principal market tuna on the natural scale (median posterior values and 95% credible intervals of the posterior distribution)

| Species | Population | Species-mean somatic growth | | Survival | | Age maturity (years) | | Spawning frequency (days) | | Spawning duration (days) | | Batch fecundity (x10^6^) | | Annual Fecundity (x10^8^) | |
| --- | --- | --- | --- | --- | --- | --- | --- | --- | --- | --- | --- | --- | --- | --- | --- |
|  |  | Med. | 95-CI | Med. | 95-CI | Med. | 95-CI | Med. | 95-CI | Med. | 95-CI | Med. | 95-CI | Med. | 95-CI |
| Skipjack tuna *Katsuwonus pelamis* | Eastern Atlantic | 0.33 | 0.22, 0.49 | 0.40 | 0.29, 0.53 | 1.80 | 0.50, 6.74 | 1.80 | 0.58, 5.31 | 364.91 | 357, 365.00 | 0.35 | 0.21, 0.70 | 1.15 | 0.61, 2.22 |
|  | Eastern Pacific | 0.61 | 0.44, 0.84 | 0.35 | 0.27, 0.44 | 1.84 | 0.44, 7.81 | 2.25 | 0.81, 6.17 | 325.43 | 23.73, 364.68 | 0.34 | 0.21, 0.61 | 1.16 | 0.61, 2.24 |
|  | Indian | 0.49 | 0.38, 0.63 | 0.38 | 0.28, 0.50 | 1.53 | 1.08, 2.17 | 1.73 | 0.63, 4.48 | 364.98 | 364.79, 365.00 | 0.35 | 0.25, 0.51 | 1.16 | 0.62, 2.22 |
|  | Western Atlantic | 0.21 | 0.15, 0.29 | 0.42 | 0.33, 0.52 | 1.97 | 1.38, 2.79 | 2.28 | 0.62, 7.79 | 256.60 | 7.28, 364.10 | 0.36 | 0.21, 0.81 | 0.16 | 0.60, 2.23 |
|  | Western Pacific | 0.67 | 0.57, 0.78 | 0.34 | 0.26, 0.43 | 1.75 | 0.44, 6.91 | 2.27 | 1.96, 2.64 | 363.50 | 324.99, 364.95 | 0.33 | 0.25, 0.43 | 1.16 | 0.61, 2.23 |
| Albacore tuna Thunnus *alalunga* | Indian | 0.15 | 0.10, 0.21 | 0.64 | 0.53, 0.75 | 4.57 | 1.24, 16.84 | 1.61 | 0.61, 4.35 | 205.78 | 0.06, 364.96 | 1.01 | 0.60, 1.77 | 1.18 | 0.86, 1.57 |
|  | Mediterranean | 0.28 | 0.20, 0.39 | 0.51 | 0.42, 0.60 | 3.57 | 0.81, 15.69 | 1.60 | 0.53, 5.19 | 329.16 | 0.15, 365.00 | 0.96 | 0.48, 1.69 | 1.18 | 0.80, 1.67 |
|  | Northern Atlantic | 0.23 | 0.19, 0.27 | 0.61 | 0.54, 0.68 | 4.46 | 1.34, 14.83 | 1.60 | 0.67, 4.02 | 230.05 | 7.37, 362.44 | 0.98 | 0.59, 1.65 | 1.18 | 0.89, 1.52 |
|  | Northern Pacific | 0.24 | 0.19, 0.30 | 0.62 | 0.52, 0.72 | 4.59 | 1.40, 15.15 | 1.67 | 1.26, 2.23 | 209.61 | 16.05, 356.12 | 0.97 | 0.67, 1.38 | 1.18 | 0.90, 1.50 |
|  | Southern Atlantic | 0.15 | 0.10, 0.24 | 0.67 | 0.58, 0.76 | 4.89 | 1.33, 17.53 | 1.58 | 0.59, 4.27 | 206.91 | 0.07, 364.95 | 0.97 | 0.67, 1.40 | 1.18 | 0.86, 1.57 |
|  | Southern Pacific | 0.19 | 0.14, 0.27 | 0.65 | 0.58, 0.72 | 4.54 | 3.24, 6.39 | 1.36 | 1.03, 1.81 | 174.19 | 30.93, 329.31 | 1.03 | 0.73, 1.52 | 1.20 | 1.16, 1.25 |
| Yellowfin tuna *Thunnus albacores* | Atlantic | 0.34 | 0.26, 0.43 | 0.47 | 0.37, 0.56 | 2.10 | 0.55, 8.22 | 3.06 | 2.21, 4.11 | 219.23 | 28.60, 353.94 | 2.37 | 1.65, 3.21 | 1.14 | 0.60, 2.17 |
|  | Eastern Pacific | 0.56 | 0.44, 0.72 | 0.41 | 0.32, 0.51 | 1.93 | 0.49, 7.81 | 1.27 | 1.03, 1.58 | 364.95 | 358.23, 365.00 | 2.26 | 1.61, 2.93 | 1.14 | 0.60, 2.22 |
|  | Indian | 0.31 | 0.23, 0.41 | 0.51 | 0.4, 0.62 | 2.29 | 0.64, 8.52 | 2.01 | 0.74, 5.22 | 276.65 | 11.49, 363.81 | 2.55 | 1.90, 3.76 | 1.14 | 0.60, 2.16 |
|  | Western Pacific | 0.35 | 0.28, 0.43 | 0.45 | 0.34, 0.56 | 2.32 | 1.63, 3.3 | 1.74 | 1.50, 2.02 | 359.61 | 318.18, 364.43 | 2.51 | 2.00, 3.24 | 1.14 | 0.62, 2.12 |
| Southern bluefin tuna *Thunnus maccoyii* | Southern | 0.15 | 0.12, 0.19 | 0.82 | 0.77, 0.86 | 10.73 | 8.29, 13.78 | 1.14 | 0.85, 1.54 | 140.50 | 6.50, 349.10 | 5.96 | 3.60, 9.87 | 1.13 | 0.77, 1.66 |
| Bigeye tuna *Thunnus obesus* | Atlantic | 0.14 | 0.12, 0.18 | 0.62 | 0.54, 0.69 | 2.15 | 0.56, 8.36 | 1.52 | 0.53, 4.62 | 351.86 | 0.80, 365.00 | 2.06 | 1.18 3.83 | 1.13 | 0.59, 2.14 |
|  | Eastern Pacific | 0.22 | 0.16, 0.32 | 0.57 | 0.46, 0.66 | 1.98 | 0.59, 6.77 | 1.32 | 1.00, 1.76 | 363.66 | 327.83, 364.95 | 1.90 | 1.23, 2.72 | 1.12 | 0.61, 2.10 |
|  | Indian | 0.25 | 0.18, 0.36 | 0.57 | 0.44, 0.68 | 1.98 | 1.4,  2.8 | 1.64 | 0.65, 4.63 | 279.95 | 10.75, 364.14 | 2.01 | 1.11, 3.51 | 1.13 | 0.60, 2.11 |
|  | Western Pacific | 0.27 | 0.19, 0.37 | 0.61 | 0.53, 0.69 | 2.20 | 1.55, 3.10 | 1.11 | 0.91, 1.37 | 364.96 | 363.26, 365.00 | 2.12 | 1.54 2.96 | 1.13 | 0.60, 2.13 |
| Pacific bluefin tuna *Thunnus orientalis* | Pacific | 0.15 | 0.10, 0.21 | 0.72 | 0.62, 0.80 | 4.06 | 2.87, 5.74 | 3.18 | 2.55, 3.91 | 111.27 | 1.61, 356.78 | 14.81 | 8.76, 24.5 | 1.12 | 0.76, 1.63 |
| Atlantic bluefin tuna *Thunnus thynnus* | Eastern Atlantic | 0.09 | 0.07, 0.12 | 0.80 | 0.72, 0.86 | 3.13 | 2.44, 4.07 | 1.25 | 0.94, 1.67 | 84.61 | 3.14, 335.15 | 8.63 | 5.20, 14.24 | 1.07 | 1.03, 1.11 |
|  | Western Atlantic | 0.13 | 0.10, 0.16 | 0.84 | 0.8, 0.87 | 10.89 | 8.82, 13.39 | 1.33 | 0.45, 3.90 | 122.99 | 1.66, 358.41 | 8.70 | 4.08, 18.62 | 1.09 | 0.78, 1.58 |
