# Supplementary material for: Global reconstruction of life‐history strategies: A case study using tunas
Source: J Appl Ecol. 2019 Feb 1;56(4):855–65. doi: 10.1111/1365-2664.13327 (PMC6559282; doi:10.1111/1365-2664.13327)
Supplement: Supplementary file 8 [file JPE-56-855-s008.docx]

**Supporting information for Horswill et al. *Global reconstruction of life-history strategies***

Table S4. The change in credible intervals (CI) associated with the reconstructed life history traits for Southern bluefin tuna, comparing a model that incorporates all available data and a model that includes somatic growth rate only.

| Southern bluefin | Full model | | | Reduced model | | | % Change |
| --- | --- | --- | --- | --- | --- | --- | --- |
|  | 2.5 CI | Median | 97.5 CI | 2.5 CI | Median | 97.5 CI |  |
| Survival | 0.80 | 1.14 | 1.47 | -1.31 | 0.30 | 1.91 | 376.05 |
| Maturity | 0.81 | 1.13 | 1.45 | -1.59 | 0.06 | 1.72 | 419.05 |
| Spawning duration | -1.38 | -0.70 | -0.02 | -1.42 | -0.69 | 0.04 | 7.71 |
| Spawning frequency | -1.69 | -1.01 | -0.29 | -2.88 | 0.13 | 3.13 | 327.66 |
| Batch fecundity | 0.68 | 1.13 | 1.59 | -2.80 | 0.45 | 3.69 | 612.43 |
| Annual fecundity | -4.60 | -0.12 | 4.40 | -4.46 | -0.06 | 4.29 | -2.78 |
| Somatic growth | -1.10 | -0.81 | -0.52 | -1.09 | -0.80 | -0.51 | 0.00 |
