# Supplementary material for: Global reconstruction of life‐history strategies: A case study using tunas
Source: J Appl Ecol. 2019 Feb 1;56(4):855–65. doi: 10.1111/1365-2664.13327 (PMC6559282; doi:10.1111/1365-2664.13327)
Supplement: Supplementary file 9 [file JPE-56-855-s009.docx]

**Supporting information for Horswill et al. *Global reconstruction of life-history strategies***

Table S5. Percentage change in the credible intervals (CI) of the life-history traits reconstructed using a model with seven life-history traits (including the four fecundity metrics) compared with traits reconstructed using a model based on three life-history traits (somatic growth, survival and age of maturity). Variables on the scale of the linear predictor.

| Trait | Species / Stock | Model with 7 traits | | | Model with 3 traits | | | % change in 95 CI |
| --- | --- | --- | --- | --- | --- | --- | --- | --- |
|  |  | 2.5 CI | median | 97.5 CI | 2.5 CI | median | 97.5 CI |  |
| Survival | Katsuwonus pelamis Eastern Atlantic stock | -1.60 | -1.05 | -0.44 | -1.54 | -1.00 | -0.40 | -0.44 |
|  | Katsuwonus pelamis Eastern Pacific stock | -1.71 | -1.29 | -0.86 | -1.73 | -1.30 | -0.88 | -0.61 |
|  | Katsuwonus pelamis Indian stock | -1.69 | -1.16 | -0.57 | -1.65 | -1.13 | -0.58 | -4.20 |
|  | Katsuwonus pelamis Western Atlantic stock | -1.39 | -0.95 | -0.49 | -1.37 | -0.93 | -0.46 | 1.69 |
|  | Katsuwonus pelamis Western Pacific stock | -1.78 | -1.35 | -0.93 | -1.79 | -1.35 | -0.93 | 1.71 |
|  | Thunnus alalunga Indian stock | -0.44 | 0.09 | 0.67 | -0.42 | 0.11 | 0.69 | 0.14 |
|  | Thunnus alalunga Mediterranean stock | -0.97 | -0.56 | -0.13 | -0.97 | -0.56 | -0.14 | -1.34 |
|  | Thunnus alalunga Northern Atlantic stock | -0.40 | -0.07 | 0.27 | -0.41 | -0.07 | 0.27 | 1.48 |
|  | Thunnus alalunga Northern Pacific stock | -0.51 | -0.02 | 0.52 | -0.54 | -0.03 | 0.54 | 4.87 |
|  | Thunnus alalunga Southern Atlantic stock | -0.23 | 0.22 | 0.76 | -0.22 | 0.25 | 0.79 | 2.65 |
|  | Thunnus alalunga Southern Pacific stock | -0.23 | 0.13 | 0.52 | -0.24 | 0.13 | 0.52 | 1.81 |
|  | Thunnus albacares Atlantic stock | -1.19 | -0.74 | -0.32 | -1.18 | -0.75 | -0.33 | -2.43 |
|  | Thunnus albacares Eastern Pacific stock | -1.45 | -1.00 | -0.55 | -1.48 | -1.03 | -0.60 | -2.51 |
|  | Thunnus albacares Indian stock | -1.03 | -0.56 | -0.04 | -1.05 | -0.57 | -0.05 | 0.69 |
|  | Thunnus albacares Western Pacific stock | -1.35 | -0.81 | -0.31 | -1.41 | -0.83 | -0.31 | 5.67 |
|  | Thunnus maccoyii Southern stock | 0.80 | 1.14 | 1.47 | 0.80 | 1.14 | 1.48 | 0.79 |
|  | Thunnus obesus Atlantic stock | -0.39 | -0.03 | 0.33 | -0.38 | -0.02 | 0.34 | -1.10 |
|  | Thunnus obesus Eastern Pacific stock | -0.77 | -0.27 | 0.19 | -0.79 | -0.27 | 0.18 | 1.82 |
|  | Thunnus obesus Indian stock | -0.86 | -0.25 | 0.29 | -0.86 | -0.27 | 0.25 | -3.20 |
|  | Thunnus obesus Western Pacific stock | -0.43 | -0.05 | 0.34 | -0.42 | -0.04 | 0.35 | 0.70 |
|  | Thunnus orientalis Pacific stock | -0.01 | 0.48 | 0.98 | -0.03 | 0.45 | 0.93 | -2.98 |
|  | Thunnus thynnus Eastern Atlantic stock | 0.52 | 1.00 | 1.46 | 0.49 | 0.98 | 1.44 | 1.14 |
|  | Thunnus thynnus Western Atlantic stock | 0.99 | 1.30 | 1.60 | 0.99 | 1.30 | 1.61 | 0.85 |
| Maturity | Katsuwonus pelamis Eastern Atlantic stock | -2.70 | -1.11 | 0.55 | -2.60 | -1.05 | 0.52 | -4.08 |
|  | Katsuwonus pelamis Eastern Pacific stock | -2.88 | -1.08 | 0.73 | -2.86 | -1.16 | 0.57 | -5.14 |
|  | Katsuwonus pelamis Indian stock | -1.75 | -1.31 | -0.87 | -1.73 | -1.30 | -0.86 | -0.60 |
|  | Katsuwonus pelamis Western Atlantic stock | -1.44 | -0.99 | -0.56 | -1.43 | -1.00 | -0.55 | -0.24 |
|  | Katsuwonus pelamis Western Pacific stock | -2.87 | -1.14 | 0.58 | -2.96 | -1.19 | 0.54 | 1.59 |
|  | Thunnus alalunga Indian stock | -1.58 | 0.06 | 1.70 | -1.49 | 0.07 | 1.63 | -4.68 |
|  | Thunnus alalunga Mediterranean stock | -2.12 | -0.25 | 1.61 | -2.11 | -0.31 | 1.56 | -1.53 |
|  | Thunnus alalunga Northern Atlantic stock | -1.48 | 0.03 | 1.54 | -1.49 | 0.00 | 1.49 | -1.26 |
|  | Thunnus alalunga Northern Pacific stock | -1.42 | 0.07 | 1.57 | -1.48 | 0.05 | 1.56 | 1.72 |
|  | Thunnus alalunga Southern Atlantic stock | -1.48 | 0.15 | 1.75 | -1.37 | 0.16 | 1.72 | -4.41 |
|  | Thunnus alalunga Southern Pacific stock | -0.37 | 0.05 | 0.48 | -0.38 | 0.05 | 0.48 | 1.00 |
|  | Thunnus albacares Atlantic stock | -2.59 | -0.91 | 0.80 | -2.50 | -0.92 | 0.65 | -7.02 |
|  | Thunnus albacares Eastern Pacific stock | -2.75 | -1.02 | 0.73 | -2.79 | -1.05 | 0.67 | -0.78 |
|  | Thunnus albacares Indian stock | -2.40 | -0.80 | 0.84 | -2.40 | -0.81 | 0.81 | -0.71 |
|  | Thunnus albacares Western Pacific stock | -1.23 | -0.79 | -0.35 | -1.22 | -0.79 | -0.35 | -1.91 |
|  | Thunnus maccoyii Southern stock | 0.81 | 1.13 | 1.45 | 0.81 | 1.13 | 1.45 | -0.08 |
|  | Thunnus obesus Atlantic stock | -2.57 | -0.88 | 0.82 | -2.50 | -0.87 | 0.74 | -4.46 |
|  | Thunnus obesus Eastern Pacific stock | -2.50 | -0.99 | 0.56 | -2.54 | -1.00 | 0.51 | -0.03 |
|  | Thunnus obesus Indian stock | -1.43 | -0.99 | -0.55 | -1.42 | -1.00 | -0.57 | -2.52 |
|  | Thunnus obesus Western Pacific stock | -1.29 | -0.86 | -0.42 | -1.28 | -0.85 | -0.41 | 0.40 |
|  | Thunnus orientalis Pacific stock | -0.52 | -0.09 | 0.35 | -0.52 | -0.08 | 0.34 | -0.86 |
|  | Thunnus thynnus Eastern Atlantic stock | -0.72 | -0.41 | -0.08 | -0.74 | -0.42 | -0.09 | 0.46 |
|  | Thunnus thynnus Western Atlantic stock | 0.89 | 1.15 | 1.41 | 0.88 | 1.15 | 1.41 | 0.47 |
| Growth | Katsuwonus pelamis Eastern Atlantic stock | -0.28 | 0.30 | 0.85 | -0.33 | 0.24 | 0.80 | 0.14 |
|  | Katsuwonus pelamis Eastern Pacific stock | 0.70 | 1.16 | 1.62 | 0.74 | 1.19 | 1.64 | -1.98 |
|  | Katsuwonus pelamis Indian stock | 0.51 | 0.86 | 1.20 | 0.49 | 0.83 | 1.18 | -1.41 |
|  | Katsuwonus pelamis Western Atlantic stock | -0.78 | -0.34 | 0.10 | -0.77 | -0.33 | 0.11 | -0.82 |
|  | Katsuwonus pelamis Western Pacific stock | 1.06 | 1.29 | 1.52 | 1.07 | 1.30 | 1.53 | -0.25 |
|  | Thunnus alalunga Indian stock | -1.34 | -0.83 | -0.33 | -1.33 | -0.84 | -0.34 | -1.88 |
|  | Thunnus alalunga Mediterranean stock | -0.44 | 0.05 | 0.55 | -0.45 | 0.06 | 0.56 | 2.32 |
|  | Thunnus alalunga Northern Atlantic stock | -0.47 | -0.22 | 0.03 | -0.48 | -0.22 | 0.03 | 1.82 |
|  | Thunnus alalunga Northern Pacific stock | -0.49 | -0.17 | 0.16 | -0.49 | -0.16 | 0.17 | 1.37 |
|  | Thunnus alalunga Southern Atlantic stock | -1.44 | -0.79 | -0.15 | -1.47 | -0.82 | -0.18 | 0.14 |
|  | Thunnus alalunga Southern Pacific stock | -0.93 | -0.45 | 0.03 | -0.92 | -0.45 | 0.04 | -0.03 |
|  | Thunnus albacares Atlantic stock | -0.03 | 0.33 | 0.67 | -0.01 | 0.34 | 0.69 | 0.14 |
|  | Thunnus albacares Eastern Pacific stock | 0.71 | 1.06 | 1.41 | 0.70 | 1.05 | 1.40 | -0.24 |
|  | Thunnus albacares Indian stock | -0.23 | 0.19 | 0.61 | -0.21 | 0.21 | 0.62 | -0.54 |
|  | Thunnus albacares Western Pacific stock | 0.09 | 0.37 | 0.66 | 0.08 | 0.38 | 0.67 | 2.69 |
|  | Thunnus maccoyii Southern stock | -1.10 | -0.81 | -0.52 | -1.10 | -0.81 | -0.52 | -0.43 |
|  | Thunnus obesus Atlantic stock | -1.18 | -0.88 | -0.57 | -1.18 | -0.87 | -0.57 | 0.21 |
|  | Thunnus obesus Eastern Pacific stock | -0.75 | -0.26 | 0.24 | -0.78 | -0.28 | 0.22 | 1.10 |
|  | Thunnus obesus Indian stock | -0.57 | -0.08 | 0.43 | -0.53 | -0.04 | 0.46 | -0.88 |
|  | Thunnus obesus Western Pacific stock | -0.45 | 0.00 | 0.46 | -0.49 | -0.03 | 0.42 | -0.18 |
|  | Thunnus orientalis Pacific stock | -1.38 | -0.86 | -0.34 | -1.36 | -0.84 | -0.33 | -0.74 |
|  | Thunnus thynnus Eastern Atlantic stock | -1.96 | -1.52 | -1.09 | -1.96 | -1.52 | -1.09 | 0.14 |
|  | Thunnus thynnus Western Atlantic stock | -1.38 | -1.04 | -0.71 | -1.38 | -1.05 | -0.70 | 1.49 |
