# Supplementary material for: Global reconstruction of life‐history strategies: A case study using tunas
Source: J Appl Ecol. 2019 Feb 1;56(4):855–65. doi: 10.1111/1365-2664.13327 (PMC6559282; doi:10.1111/1365-2664.13327)
Supplement: Supplementary file 10 [file JPE-56-855-s010.docx]

**Supporting information for Horswill et al. *Global reconstruction of life-history strategies***

# Appendix S1: Code for model of life-history traits

# Fitted in JAGS: package ”jagsUI” v1.4.9

writeLines("

writeLines("

model{

#Priors and constraints

beta2[1]~dnorm(0,0.1)

beta2[2]~dnorm(0,0.1)

beta2[3]~dnorm(0,0.1)

beta2[4]~dnorm(0,0.1)

beta2[5]~dnorm(0,0.1)

beta2[6]~dnorm(0,0.1)

beta2[7]~dnorm(0,0.1)

Sigma[1:(ntraits),1:(ntraits)] <- inverse(Tau_mat[,])

Tau_mat[1:(ntraits),1:(ntraits)] ~ dwish(Omega[,],(ntraits)+1)

for(i in 1:(ntraits)) {

xi[i]~dunif(0,5)

xi1[i]~dunif(0,5)

}

for (i in 1:ntraits){

mu_raw[i]~dnorm(0,15)

mu_raw1[i]~dnorm(0,50)

}

Sigma1[1:(ntraits),1:(ntraits)] <- inverse(Tau_mat1[,])

Tau_mat1[1:(ntraits),1:(ntraits)] ~ dwish(Omega1[,],(ntraits)+1)

species_prec[1] ~ dnorm(20,0.1)T(0.0,)

species_prec[2] ~ dnorm(20,0.1)T(0.0,)

species_prec[3] ~ dnorm(20,0.1)T(0.0,)

species_prec[4] ~ dnorm(20,0.1)T(0.0,)

species_prec[5] ~ dnorm(20,0.1)T(0.0,)

species_prec[6] ~ dnorm(20,0.1)T(0.0,)

species_prec[7] ~ dnorm(20,0.1)T(0.0,)

# __________ Observation component ______________________

Spawn_d[1]~dnorm(mu[1,1],species_prec[1])

Spawn_d[2]~dnorm(mu[2,1],species_prec[1])

Spawn_d[3]~dnorm(mu[3,1],species_prec[1])

Spawn_d[4]~dnorm(mu[3,1],species_prec[1])

Spawn_d[5]~dnorm(mu[3,1],species_prec[1])

Spawn_d[6]~dnorm(mu[3,1],species_prec[1])

Spawn_d[7]~dnorm(mu[3,1],species_prec[1])

Spawn_d[8]~dnorm(mu[4,1],species_prec[1])

Spawn_d[9]~dnorm(mu[5,1],species_prec[1])

Spawn_d[10]~dnorm(mu[5,1],species_prec[1])

Spawn_d[11]~dnorm(mu[8,1],species_prec[1])

Spawn_d[12]~dnorm(mu[9,1],species_prec[1])

Spawn_d[13]~dnorm(mu[9,1],species_prec[1])

Spawn_d[14]~dnorm(mu[11,1],species_prec[1])

Spawn_d[15]~dnorm(mu[11,1],species_prec[1])

Spawn_d[16]~dnorm(mu[11,1],species_prec[1])

Spawn_d[17]~dnorm(mu[11,1],species_prec[1])

Spawn_d[18]~dnorm(mu[11,1],species_prec[1])

Spawn_d[19]~dnorm(mu[12,1],species_prec[1])

Spawn_d[20]~dnorm(mu[12,1],species_prec[1])

Spawn_d[21]~dnorm(mu[12,1],species_prec[1])

Spawn_d[22]~dnorm(mu[13,1],species_prec[1])

Spawn_d[23]~dnorm(mu[14,1],species_prec[1])

Spawn_d[24]~dnorm(mu[15,1],species_prec[1])

Spawn_d[25]~dnorm(mu[15,1],species_prec[1])

Spawn_d[26]~dnorm(mu[15,1],species_prec[1])

Spawn_d[27]~dnorm(mu[15,1],species_prec[1])

Spawn_d[28]~dnorm(mu[15,1],species_prec[1])

Spawn_d[29]~dnorm(mu[16,1],species_prec[1])

Spawn_d[30]~dnorm(mu[16,1],species_prec[1])

Spawn_d[31]~dnorm(mu[18,1],species_prec[1])

Spawn_d[32]~dnorm(mu[18,1],species_prec[1])

Spawn_d[33]~dnorm(mu[19,1],species_prec[1])

Spawn_d[34]~dnorm(mu[20,1],species_prec[1])

Spawn_d[35]~dnorm(mu[20,1],species_prec[1])

Spawn_d[36]~dnorm(mu[21,1],species_prec[1])

Spawn_d[37]~dnorm(mu[22,1],species_prec[1])

Spawn_d[38]~dnorm(mu[22,1],species_prec[1])

Spawn_d[39]~dnorm(mu[23,1],species_prec[1])

Phi[1]~dnorm(mu[1,2], species_prec[2])

for (i in 2:4){

Phi[i]~dnorm(mu[2,2], species_prec[2]) }

Phi[5]~dnorm(mu[3,2], species_prec[2])

for (i in 6:8){

Phi[i]~dnorm(mu[4,2], species_prec[2]) }

for (i in 9:11){

Phi[i]~dnorm(mu[5,2], species_prec[2]) }

Phi[12]~dnorm(mu[6,2], species_prec[2])

for (i in 13:17){

Phi[i]~dnorm(mu[7,2], species_prec[2]) }

for (i in 18:22){

Phi[i]~dnorm(mu[8,2], species_prec[2]) }

Phi[23]~dnorm(mu[9,2], species_prec[2])

for (i in 24:25){

Phi[i]~dnorm(mu[10,2], species_prec[2]) }

for (i in 26:29){

Phi[i]~dnorm(mu[11,2], species_prec[2]) }

for (i in 30:32){

Phi[i]~dnorm(mu[12,2], species_prec[2]) }

for (i in 33:35){

Phi[i]~dnorm(mu[13,2], species_prec[2]) }

for (i in 36:37){

Phi[i]~dnorm(mu[14,2], species_prec[2]) }

Phi[38]~dnorm(mu[15,2], species_prec[2])

for (i in 39:45){

Phi[i]~dnorm(mu[16,2], species_prec[2]) }

for (i in 46:50){

Phi[i]~dnorm(mu[17,2], species_prec[2]) }

for (i in 51:52){

Phi[i]~dnorm(mu[18,2], species_prec[2]) }

Phi[53]~dnorm(mu[19,2], species_prec[2])

for (i in 54:57){

Phi[i]~dnorm(mu[20,2], species_prec[2]) }

for (i in 58:60){

Phi[i]~dnorm(mu[21,2], species_prec[2]) }

for (i in 61:63){

Phi[i]~dnorm(mu[22,2], species_prec[2]) }

for (i in 64:71){

Phi[i]~dnorm(mu[23,2], species_prec[2]) }

m[1]~dnorm(mu[3,3], species_prec[3])

m[2]~dnorm(mu[4,3], species_prec[3])

m[3]~dnorm(mu[11,3], species_prec[3])

m[4]~dnorm(mu[15,3], species_prec[3])

m[5]~dnorm(mu[16,3], species_prec[3])

m[6]~dnorm(mu[16,3], species_prec[3])

m[7]~dnorm(mu[19,3], species_prec[3])

m[8]~dnorm(mu[20,3], species_prec[3])

m[9]~dnorm(mu[21,3], species_prec[3])

m[10]~dnorm(mu[22,3], species_prec[3])

m[11]~dnorm(mu[22,3], species_prec[3])

m[12]~dnorm(mu[23,3], species_prec[3])

m[13]~dnorm(mu[23,3], species_prec[3])

m[14]~dnorm(mu[23,3], species_prec[3])

Fec[1]~dnorm(mu[3,4], species_prec[4])

Fec[2]~dnorm(mu[5,4], species_prec[4])

Fec[3]~dnorm(mu[5,4], species_prec[4])

Fec[4]~dnorm(mu[5,4], species_prec[4])

Fec[5]~dnorm(mu[9,4], species_prec[4])

Fec[6]~dnorm(mu[10,4], species_prec[4])

Fec[7]~dnorm(mu[11,4], species_prec[4])

Fec[8]~dnorm(mu[12,4], species_prec[4])

Fec[9]~dnorm(mu[13,4], species_prec[4])

Fec[10]~dnorm(mu[13,4], species_prec[4])

Fec[11]~dnorm(mu[14,4], species_prec[4])

Fec[12]~dnorm(mu[15,4], species_prec[4])

Fec[13]~dnorm(mu[15,4], species_prec[4])

Fec[14]~dnorm(mu[15,4], species_prec[4])

Fec[15]~dnorm(mu[16,4], species_prec[4])

Fec[16]~dnorm(mu[18,4], species_prec[4])

Fec[17]~dnorm(mu[20,4], species_prec[4])

Fec[18]~dnorm(mu[20,4], species_prec[4])

Fec[19]~dnorm(mu[21,4], species_prec[4])

Fec[20]~dnorm(mu[22,4], species_prec[4])

Spawn_f[1]~dnorm(mu[5,5], species_prec[5])

Spawn_f[2]~dnorm(mu[5,5], species_prec[5])

Spawn_f[3]~dnorm(mu[5,5], species_prec[5])

Spawn_f[4]~dnorm(mu[5,5], species_prec[5])

Spawn_f[5]~dnorm(mu[9,5], species_prec[5])

Spawn_f[6]~dnorm(mu[11,5], species_prec[5])

Spawn_f[7]~dnorm(mu[12,5], species_prec[5])

Spawn_f[8]~dnorm(mu[13,5], species_prec[5])

Spawn_f[9]~dnorm(mu[13,5], species_prec[5])

Spawn_f[10]~dnorm(mu[15,5], species_prec[5])

Spawn_f[11]~dnorm(mu[15,5], species_prec[5])

Spawn_f[12]~dnorm(mu[15,5], species_prec[5])

Spawn_f[13]~dnorm(mu[15,5], species_prec[5])

Spawn_f[14]~dnorm(mu[16,5], species_prec[5])

Spawn_f[15]~dnorm(mu[18,5], species_prec[5])

Spawn_f[16]~dnorm(mu[20,5], species_prec[5])

Spawn_f[17]~dnorm(mu[20,5], species_prec[5])

Spawn_f[18]~dnorm(mu[21,5], species_prec[5])

Spawn_f[19]~dnorm(mu[21,5], species_prec[5])

Spawn_f[20]~dnorm(mu[22,5], species_prec[5])

annual_fec[1]~dnorm(mu[11,6], species_prec[6])

annual_fec[2]~dnorm(mu[22,6], species_prec[6])

for (i in 1:3){

k[i]~dnorm(mu[1,7], species_prec[7]) }

for (i in 4:8){

k[i]~dnorm(mu[2,7], species_prec[7]) }

for (i in 9:17){

k[i]~dnorm(mu[3,7], species_prec[7]) }

for (i in 18:23){

k[i]~dnorm(mu[4,7], species_prec[7]) }

for (i in 24:45){

k[i]~dnorm(mu[5,7], species_prec[7]) }

for (i in 46:49){

k[i]~dnorm(mu[6,7], species_prec[7]) }

for (i in 50:53){

k[i]~dnorm(mu[7,7], species_prec[7]) }

for (i in 54:71){

k[i]~dnorm(mu[8,7], species_prec[7]) }

for (i in 72:81){

k[i]~dnorm(mu[9,7], species_prec[7]) }

for (i in 82:83){

k[i]~dnorm(mu[10,7], species_prec[7]) }

for (i in 84:87){

k[i]~dnorm(mu[11,7], species_prec[7]) }

for (i in 88:96){

k[i]~dnorm(mu[12,7], species_prec[7]) }

for (i in 97:105){

k[i]~dnorm(mu[13,7], species_prec[7]) }

for (i in 106:111){

k[i]~dnorm(mu[14,7], species_prec[7]) }

for (i in 112:124){

k[i]~dnorm(mu[15,7], species_prec[7]) }

for (i in 125:138){

k[i]~dnorm(mu[16,7], species_prec[7]) }

for (i in 139:150){

k[i]~dnorm(mu[17,7], species_prec[7]) }

for (i in 151:154){

k[i]~dnorm(mu[18,7], species_prec[7]) }

for (i in 155:158){

k[i]~dnorm(mu[19,7], species_prec[7]) }

for (i in 159:163){

k[i]~dnorm(mu[20,7], species_prec[7]) }

for (i in 164:167){

k[i]~dnorm(mu[21,7], species_prec[7]) }

for (i in 168:173){

k[i]~dnorm(mu[22,7], species_prec[7]) }

for (i in 174:183){

k[i]~dnorm(mu[23,7], species_prec[7]) }

# __________ Demographic trait functions ______________

for (l in 1:(ntraits)){

mu[1,l]<- gamma[1,l] + alpha[1,l] + beta2[l]*H[1]

mu[2,l]<- gamma[1,l] + alpha[2,l] + beta2[l]*H[1]

mu[3,l]<- gamma[1,l] + alpha[3,l] + beta2[l]*H[1]

mu[4,l]<- gamma[1,l] + alpha[4,l] + beta2[l]*H[1]

mu[5,l]<- gamma[1,l] + alpha[5,l] + beta2[l]*H[1]

mu[6,l]<- gamma[2,l] + alpha[6,l] + beta2[l]*H[2]

mu[7,l]<- gamma[2,l] + alpha[7,l] + beta2[l]*H[2]

mu[8,l]<- gamma[2,l] + alpha[8,l] + beta2[l]*H[2]

mu[9,l]<- gamma[2,l] + alpha[9,l] + beta2[l]*H[2]

mu[10,l]<- gamma[2,l] + alpha[10,l] + beta2[l]*H[2]

mu[11,l]<- gamma[2,l] + alpha[11,l] + beta2[l]*H[2]

mu[12,l]<- gamma[3,l] + alpha[12,l] + beta2[l]*H[3]

mu[13,l]<- gamma[3,l] + alpha[13,l] + beta2[l]*H[3]

mu[14,l]<- gamma[3,l] + alpha[14,l] + beta2[l]*H[3]

mu[15,l]<- gamma[3,l] + alpha[15,l] + beta2[l]*H[3]

mu[16,l]<- gamma[4,l] + alpha[16,l] + beta2[l]*H[4]

mu[17,l]<- gamma[5,l] + alpha[17,l] + beta2[l]*H[5]

mu[18,l]<- gamma[5,l] + alpha[18,l] + beta2[l]*H[5]

mu[19,l]<- gamma[5,l] + alpha[19,l] + beta2[l]*H[5]

mu[20,l]<- gamma[5,l] + alpha[20,l] + beta2[l]*H[5]

mu[21,l]<- gamma[6,l] + alpha[21,l] + beta2[l]*H[6]

mu[22,l]<- gamma[7,l] + alpha[22,l] + beta2[l]*H[7]

mu[23,l]<- gamma[7,l] + alpha[23,l] + beta2[l]*H[7]

}

# __________ Species loop ______________________

for (j in 1:nspecies){

E0_raw[j,1:(ntraits)] ~ dmnorm(mu_raw[],Tau_mat[,])

for (m in 1:(ntraits)){

gamma[j,m] <- xi[m]*E0_raw[j,m]}}

# __________ Population loop ______________________

for (j in 1:(nstocks)){

E0_raw1[j,1:(ntraits)] ~ dmnorm(mu_raw1[],Tau_mat1[,])

for (m in 1:(ntraits)){

alpha[j,m] <- xi1[m]*E0_raw1[j,m]}}

} ", con="model.txt")
