# Supplementary material for: Global reconstruction of life‐history strategies: A case study using tunas
Source: J Appl Ecol. 2019 Feb 1;56(4):855–65. doi: 10.1111/1365-2664.13327 (PMC6559282; doi:10.1111/1365-2664.13327)
Supplement: Supplementary file 11 [file JPE-56-855-s011.docx]

**Supporting information for Horswill et al. *Global reconstruction of life-history strategies***

# Appendix S2: Life history data for the Principal market tunas. Compiled from Juan-Jorda et al. (2016), also see Table S1

k<-c(-1.38230234,-1.180907531,-1.133203733,-0.84397007,-0.446287103,-0.261364764,-0.187535124,-0.187535124,-1.514127733,-0.891598119,-0.798507696,-0.755022584,-0.713349888,-0.693147181,-0.509160344,-0.430782916,-0.020202707,-2.302585093,-2.207274913,-1.692819521,-1.63475572,-1.237874356,-1.078809661,-1.237874356,-1.197328262,-0.916290732,-0.839329691,-0.776528789,-0.693147181,-0.597837001,-0.597837001,-0.597837001,-0.510825624,-0.287682072,-0.261364764,-0.261364764,-0.248461359,-0.056570351,-0.051293294,-0.051293294,0.09531018,0.09531018,0.223143551,0.223143551,0.262364264,-2.216407397,-2.137070655,-1.838851077,-1.820158944,-1.973281346,-1.609437912,-0.900663476,-0.616186139,-2.120263536,-2.047942875,-1.958995389,-1.771956842,-1.672910335,-1.660731207,-1.660731207,-1.609437912,-1.565421027,-1.527857925,-1.294627173,-1.272965676,-1.272965676,-1.251763468,-1.184170177,-1.139434283,-1.108662625,-0.30788478,-2.207274913,-1.771956842,-1.714798428,-1.614450454,-1.491654877,-1.30933332,-1.272965676,-1.177655496,-1.078809661,-0.494296322,-2.071473372,-1.966112856,-2.563949857,-2.120263536,-1.456716825,-0.371063681,-1.973281346,-1.320506621,-1.26940061,-1.157089218,-0.960242619,-0.867500568,-0.841647189,-0.814185509,-0.585190039,-0.653926467,-0.585190039,-0.572701027,-0.572701027,-0.525939262,-0.517514612,-0.510825624,-0.415515444,-0.415515444,-1.737271284,-1.237874356,-1.203972804,-1.080281332,-0.94160854,-0.916290732,-1.725971729,-1.609437912,-1.386294361,-1.386294361,-1.237874356,-1.231001477,-1.108662625,-0.867500568,-0.84397007,-0.789658081,-0.671385689,-0.572701027,-0.410980289,-2.244316185,-2.225624052,-2.055725015,-1.966112856,-1.966112856,-1.924148657,-1.903808973,-1.897119985,-1.832581464,-1.771956842,-1.692819521,-1.687399454,-1.676646662,-1.660731207,-2.918771232,-2.465104022,-2.26336438,-2.183025858,-2.040220829,-1.903808973,-1.832581464,-1.790360448,-1.754463684,-1.714798428,-1.46967597,-1.46967597,-2.538307427,-1.576970722,-1.139434283,-0.967584026,-1.766091722,-1.210661792,-1.177655496,-1.139434283,-1.604450371,-1.38230234,-1.378326191,-1.052683357,-1.002393431,-2.268183666,-2.120263536,-1.754463684,-1.714798428,-2.688247574,-2.659260037,-2.525728644,-2.375155786,-2.353878387,-2.302585093,-2.538307427,-2.538307427,-2.419118909,-2.407945609,-2.154165088,-2.154165088,-1.771956842,-1.62964062,-1.609437912,-1.203972804)

k2 <- (k-mean(k))/sd(k)

Phi<-c(-0.309806043,-0.657516324,-0.657516324,-0.657516324,-0.309806043,-0.657516324,-0.309806043,-0.046498493,-1.270176095,-0.882256298,-0.046498493,0.62127774,-0.657516324,-0.657516324,-0.046498493,0.164478627,0.164478627,0.164478627,0.340067608,0.557839041,0.557839041,0.62127774,0.62127774,0.93639519,0.93639519,0.490227377,0.490227377,0.93639519,1.022943267,-0.223846411,-0.214703327,0.064249252,-0.882256298,-0.470075308,-0.046498493,-0.046498493,0.490227377,-0.470075308,-0.046498493,1.102680113,1.481780096,1.87005982,2.121858717,2.121858717,2.202059753,0.164478627,0.490227377,0.490227377,0.507550982,0.841780558,-0.657516324,0.93639519,0.164478627,0.255962358,0.490227377,0.62127774,1.102680113,-0.046498493,1.102680113,1.715649798,1.022943267,1.370532854,1.533108894,1.309935591,1.427691951,1.427691951,1.673028439,1.905215789,1.939181133,1.97203367,2.034675635)

Phi2 <- (Phi-mean(Phi))/sd(Phi)

Fec<- c(12.80511929,13.32937755,12.47221362,12.24200913,13.72667934,13.65299163,13.99783211,14.5574479,14.26658618,14.73262735,14.93718812,14.58561878,15.05533302,14.81245919,15.60727003,14.18808001,14.58702247,14.80323231,16.58809928,15.99026228)

Fec2<-(Fec-mean(Fec))/sd(Fec)

Spawn_f<-c(0.631271777,0.58221562,1.490654376,0.609765572,0.530628251,0.262364264,1.208960346,0.182321557,0.2390169,0.58221562,0.688134639,0.431782416,0.500775288,0.09531018,0.262364264,0.086177696,0.09531018,1.190887565,1.193922468,0.182321557)

Spawn_f2<-(Spawn_f-mean(Spawn_f))/sd(Spawn_f)

Spawn_d<-c(365.00,152.08,365.00,365.00,365.00,365.00,365.00,121.67,182.50,365.00,182.50,152.08,212.92,121.67,121.67,121.67,182.50,212.92,,91.25,152.08,273.75,365.00,152.08,152.08,182.50,212.92,365.00,365.00,91.25,121.67,273.75,365.00,91.25,365.00,365.00,91.25,45.63,60.83,91.25)

Spawn_d[Spawn_d==365.00] <- 364.99

Spawn_d2<-Spawn_d/365

Spawn_d2.1<-log(Spawn_d2/(1-Spawn_d2)) #logit

Spawn_d3<-(Spawn_d2.1-mean(Spawn_d2.1))/sd(Spawn_d2.1)

m<- c(0.405465108,0.693147181,1.504077397,0.875468737,2.397895273,2.442347035,0.678033543,0.78845736,1.386294361,1.098612289,1.098612289,2.079441542,2.442347035,2.76000994)

m2<-(m-mean(m))/sd(m)

annual_fec<- c(18.6101,18.48889)

annual_fec2<-(annual_fec-mean(annual_fec))/sd(annual_fec)

jags.data <- list(nstocks = 23, nspecies = 7, ntraits = 7,

Omega=diag(7),Omega1=diag(7),

#Habitat: 1-tropical; 0-temperate

H=c(1,0,1,0,1,0,0),

#Somatic growth

k=k2,

#Adult Survival

Phi=Phi2,

#Fecundity

Fec=Fec2,

#Spawning freq

Spawn_f=Spawn_f2,

#Spawning duration

Spawn_d=Spawn_d3,

#Age maturity

m=m2,

#annual fecundity

annual_fec=annual_fec2)
