# Supplementary material for: Global reconstruction of life‐history strategies: A case study using tunas
Source: J Appl Ecol. 2019 Feb 1;56(4):855–65. doi: 10.1111/1365-2664.13327 (PMC6559282; doi:10.1111/1365-2664.13327)
Supplement: Supplementary file 12 [file JPE-56-855-s012.docx]

**Supporting information for Horswill et al. *Global reconstruction of life-history strategies***

**Appendix S3. Initial model structure**

During initial model development three different model structures were created. Each one included a different body metric; somatic growth rate (*K*), asymptotic body size (*L_∞_*) or maximum observed body size (*L_max_*), alongside the remaining six life-history traits (Φ*,A,S,D,F,V*). Data were collated from Juan-Jordá et al. (2016) supplemented by additional studies for batch and annual fecundity (supp. info. T1.), limiting data points to those representing females when sex information was available. The fit of these three models was compared based on the Brooks-Gelman-Rubin diagnostic tool for each parameter. These goodness of fit criteria indicated poorer mixing of the MCMC in the models that included asymptotic body size and maximum observed body size (min. effective sample size for model including *L_∞_* = 2512, all $\hat{r}$≤ 2.00; min. effective sample size for model including *L_max_* = 2186, all $\hat{r}$≤ 1.05). The model that best described the data incorporated somatic growth rate (min. effective sample size for model including *K* = 3764, all $\hat{r}$≤1.01). Consequently, the results presented relate to the model that included growth rate. The models based on the other two variables followed largely identical structures.

Body size is often viewed as the principal driver of life-histories (Blueweiss et al. 1978; Sibly & Brown 2007). However, correlations constructed with maximum body size are likely to include larger residual error introduced by single individuals. In contrast, the estimation of asymptotic size and growth rate from the von Bertalanffy growth curve typically requires large sample sizes. The von Bertalanffy growth rate is an emergent property of physiology (foraging efficiency) and behaviour (anti-predator behaviour) (Mangel 2006), and appears, at least in this context, to provide greater predictive power for estimating life-history traits, when compared to asymptotic size. This finding is in agreement with Jensen (1996) and Juan-Jordá et al. (2015). In particular, the latter of these studies reports that time-related traits, such as growth rate, rather than size-related traits, such as maximum size, better explain rates of population decline for tuna and their relatives after accounting for fishing mortality.

Blueweiss, L., Fox, H., Kudzma, V., Nakashima, D., Peters, R. & Sams, S. (1978). Relationships between body size and some life history parameters. Oecologia, 37, 257–272.

Jensen, A.L. (1996). Beverton and Holt life history invariants result from optimal trade-off of reproduction and survival. Can. J. Fish. Aquat. Sci., 53, 820–822.

Juan-Jordá, M.J., Mosqueira, I., Freire, J. & Dulvy, N.K. (2015). Population declines of tuna and relatives depend on their speed of life. Proc. R. Soc. B Biol. Sci., 282, 20150322.

Juan-Jordá, M.J., Mosqueira, I., Freire, J., Ferrer-Jordá, E. & Dulvy, N.K. (2016). Global scombrid life history data set. Ecology, 97, 809.

Mangel, M. (2006). The Theoretical Biologist’s Toolbox. Cambridge University Press, Cambridge.

Sibly, R.M. & Brown, J.H. (2007). Effects of body size and lifestyle on evolution of mammal life histories. Proc. Natl. Acad. Sci., 104, 17707–17712.
