# Supplementary material for: Global reconstruction of life‐history strategies: A case study using tunas
Source: J Appl Ecol. 2019 Feb 1;56(4):855–65. doi: 10.1111/1365-2664.13327 (PMC6559282; doi:10.1111/1365-2664.13327)
Supplement: Supplementary file 13 [file JPE-56-855-s013.docx]

**Supporting information for Horswill et al. *Global reconstruction of life-history strategies***

**Appendix S4. From total fecundity of the population to mass specific fecundity**

As described in the main text, in order to compute steepness, and the related management quantities, we require mass specific fecundity $\delta$ but the methods described in the main text produce total annual fecundity. We now show how to determine $\delta$ from total fecundity.

To do so, we use a standard age structured population model with constant rate of natural mortality $M$ (e.g. Mangel 2006, Kindsvater et al 2016). We let $\bar{N}(a)$ denote the steady state number of individuals of age *a*, with *a*=0 denoting the recruited class. Then for *a*>0 we have $\bar{N}(a)=\bar{N}(0)e^{-Ma}$ and for *a*=0

(A.1)

where, as in the main text, is the survival through the egg and larval stage to recruitment and $\bar{E}$ is the steady stage production of eggs by the population. It is given by

$\bar{E}=\sum_{a} \bar{N}(a)\delta p_{m}(a)W(a)$ (A.2)

Since $\bar{N}(a)=\bar{N}(0)e^{-Ma}$ and $\delta$ is a constant with respect to *a*, we have

$\bar{E}=\delta\bar{N}(0)\sum_{a} e^{-Ma}p_{m}(a)W(a)=\delta\bar{N}(0)\bar{W}$ (A.3)

where, as in the main text, $\bar{W}$ is the average biomass of a spawning female.

The total steady state population size $\bar{N}_{T}$ is given by

$\bar{N}_{T}=\sum_{a} \bar{N}(a)=\sum_{a} \bar{N}(0)e^{-Ma}=\bar{N}(0)\sum_{a} e^{-Ma}=\bar{N}(0)\left[\frac{1-e^{MA_{\max}}}{1-e^{-M}}\right]$ (A.4)

where, as in the main text, $A_{\max}$ is maximum age. Thus, if we know total fecundity and total population size – both predicted by our methods, we are able to compute mass specific fecundity from Eqns A.3 and A.4 since

$\delta=\frac{1}{\bar{W}}\left[\frac{1-e^{MA_{\max}}}{1-e^{-M}}\right]\frac{\bar{E}}{\bar{N}_{T}}$ (A.5)

Knowing steepness provides information about various quantities relevant to fisheries management (Mangel et al., 2013). For example, for fast-growing species (which many tunas are), the fishing mortality that gives maximum sustainable yield ($F_{MSY}$) is well approximated by $\frac{F_{MSY}}{M}=\sqrt{\frac{4h}{1-h}}-1$. The Spawning Potential Ratio at maximum sustainable yield ($SPR_{MSY}$) is defined to be lifetime offspring production of an individual when the population is fished at $F_{MSY}$ divided by lifetime offspring production of an individual in an unfished population (see Kindsvater, et al. 2016 for review). For a fast growing species, it is well approximated by $SPR_{MSY}=\sqrt{\frac{1-h}{4h}}$ (Mangel et al., 2013).

Mangel, M. *The Theoretical Biologist’s Toolbox*. (Cambridge University Press, 2006).

Mangel, M., MacCall, A.D., Brodziak, J., Dick, E., Forrest, R.E., Pourzard, R., & Ralston, S. (2013). A perspective on steepness, reference points, and stock assessment. Canadian Journal of Fisheries and Aquatic Sciences, 940, 930–940.

Kindsvater, H. K., Mangel, M., Reynolds, J. D. & Dulvy, N. K. Ten principles from evolutionary ecology essential for effective marine conservation. *Ecol. Evol.* **6,** 2125–2138 (2016).
